# Barriers to malaria prevention among immigrant travelers in the United States who visit friends and relatives in sub-Saharan Africa: A cross-sectional, multi-setting survey of knowledge, attitudes, and practices

**DOI:** 10.1371/journal.pone.0229565

**Published:** 2020-03-12

**Authors:** Hannah R. Volkman, Emily J. Walz, Danushka Wanduragala, Elizabeth Schiffman, Anne Frosch, Jonathan D. Alpern, Patricia F. Walker, Kristina M. Angelo, Christina Coyle, Mimi A. Mohamud, Esther Mwangi, Joseline Haizel-Cobbina, Comfort Nchanji, Rebecca S. Johnson, Baninla Ladze, Stephen J. Dunlop, William M. Stauffer

**Affiliations:** 1 Department of Medicine, University of Minnesota, Minneapolis, MN, United States of America; 2 Department of Veterinary and Biomedical Sciences, University of Minnesota, St Paul, MN, United States of America; 3 Minnesota Department of Health, St Paul, MN, United States of America; 4 Hennepin Healthcare, Minneapolis, MN, United States of America; 5 HealthPartners Institute, Bloomington, MN, United States of America; 6 Centers for Disease Control and Prevention, Atlanta, GA, United States of America; 7 Department of Medicine, Albert Einstein College of Medicine, New York, NY, United States of America; 8 Sierra Leone Community in Minnesota, Minneapolis, MN, United States of America; 9 Minnesota Cameroon Community, Minneapolis, MN, United States of America; Instituto Rene Rachou, BRAZIL

## Abstract

**Background:**

Despite achievements in the reduction of malaria globally, imported malaria cases to the United States by returning international travelers continue to increase. Immigrants to the United States from sub-Saharan Africa (SSA) who then travel back to their homelands to visit friends and relatives (VFRs) experience a disproportionate burden of malaria illness. Various studies have explored barriers to malaria prevention among VFRs and non-VFRs–travelers to the same destinations with other purpose for travel–but few employed robust epidemiologic study designs or performed comparative analyses of these two groups. To better quantify the key barriers that VFRs face to implement effective malaria prevention measures, we conducted a comprehensive community-based, cross-sectional, survey to identify differences in malaria prevention knowledge, attitudes, and practices (KAP) among VFRs and others traveling to Africa and describe the differences between VFRs and other types of international travelers.

**Methods and findings:**

Three distinct populations of travelers with past or planned travel to malaria-endemic countries of SSA were surveyed: VFRs diagnosed with malaria as reported through a state health department; members of the general VFR population (community); and VFR and non-VFR travelers presenting to a travel health clinic, both before their pretravel consultation and again, after return from travel. A Community Advisory Board of African immigrants and prior qualitative research informed survey development and dissemination. Across the three groups, 489 travelers completed surveys: 351 VFRs and 138 non-VFRs. VFRs who reported taking antimalarials on their last trip rated their concern about malaria higher than those who did not. Having taken five or more trips to SSA was reported more commonly among VFRs diagnosed with malaria than community VFRs (44.0% versus 20.4%; p = 0.008). Among travel health clinic patients surveyed before and after travel, VFR travelers were less successful than non-VFRs in adhering to their planned use of antimalarials (82.2% versus 98.7%; p = 0.001) and employing mosquito bite avoidance techniques (e.g., using bed nets: 56.8% versus 81.8%; p = 0.009). VFRs who visited the travel health clinic were more likely than VFR respondents from the community to report taking an antimalarial (83.0% versus 61.9%; p = 0.009), or to report bite avoidance behaviors (e.g., staying indoors when mosquitoes were out: 80.9% versus 59.5%; p = 0.009).

**Conclusions:**

We observed heterogeneity in malaria prevention behaviors among VFRs and between VFR and non-VFR traveler populations. Although VFRs attending the travel health clinic appear to demonstrate better adherence to malaria prevention measures than VFR counterparts surveyed in the community, specialized pretravel care is not sufficient to ensure chemoprophylaxis use and bite avoidance behaviors among VFRs. Even when seeking specialized pretravel care, VFRs experience greater barriers to the use of malaria prevention than non-VFRs. Addressing access to health care and upstream barrier reduction strategies that make intended prevention more achievable, affordable, easier, and resonant among VFRs may improve malaria prevention intervention effectiveness.

## Introduction

Despite global achievements in reduction of malaria morbidity and mortality [1], numbers of malaria cases imported to the United States by returning international travelers continue to increase [2]. Among reported malaria cases with a known reason for travel, immigrant travelers who visit friends and relatives (VFRs) comprised 72% of cases among US civilians in 2016 [2]. Immigrants who return to their countries of origin for the purpose of visiting friends and relatives have also contributed to the increase in malaria cases due to an increasing number and proportion of travelers who are VFRs [3,4]. Travel to the African continent was reported by 86% of malaria cases imported to the United States in 2016; travel to West Africa specifically was reported by 53% of cases [2]. Malaria is the most common reason for post-travel hospitalization among travelers [5], costing on average $25,789 per admission [6]. The recent discontinuation of intravenous quinidine and challenges with access to intravenous artesunate in the United States (it is only available through an investigative new drug protocol from CDC) only underscore the importance of malaria prevention in travelers [2].

Compared to other travelers to the same destinations VFRs have a disproportionately high prevalence of travel-related morbidity, particularly infectious diseases [7], including malaria. Among international travelers originating in developed countries, VFRs are less successful at implementing key approaches to malaria prevention than other categories of travelers to malaria endemic regions [8–11]. Additionally, VFRs tend to travel for longer periods of time than non-VFRs [8] and to countries and regions of greater malaria risk than non-VFRs [8,12]. Because they may identify both with their place of birth and current residence [13], VFRs may experience cultural dissonance when it comes to applying malaria prevention measures; for example, concern over inconveniencing one’s hosts or being judged as too Westernized by family or friends may prevent VFRs from adhering to recommended malaria prevention methods [13–15]. Although a potential challenge for travel health providers, taking into account cultural barriers when advising this population may increase the likelihood of adherence to prevention measures [13,16].

Health disparities and poor health outcomes among immigrant populations are associated with limited English proficiency and discordant languages between providers and immigrant patients [17–19], racism and lack of cultural diversity among providers [20], poor health literacy correlated to poverty [18], lack of insurance coverage [20, 21], and citizenship status [18, 21]. Barriers to health care and lack of access to preventive health services have been described for immigrant populations, but few studies have investigated barriers to receiving travel medicine services and prevention of malaria among VFRs. Various studies have explored malaria prevention among immigrants and non-immigrants but do not include comparative analyses to understand the differences among and within these groups [11, 22, 23, 24]. Anecdotal reports of VFR travel risk factors have informed the field of travel medicine [25], but epidemiologic analyses are limited in size and scope [15, 25, 26, 27].

To better characterize key barriers to effective malaria prevention among VFRs, we conducted a comprehensive community-based, cross-sectional, survey of US travelers to malaria-endemic countries in sub-Saharan Africa (SSA) informed by earlier qualitative research and community engagement [28]. This study permitted us to identify differences in malaria prevention knowledge, attitudes, and practices (KAP) among VFRs traveling to Africa and to describe differences between VFRs and non-VFRs.

## Methods

### Study design

**Fig 1:**
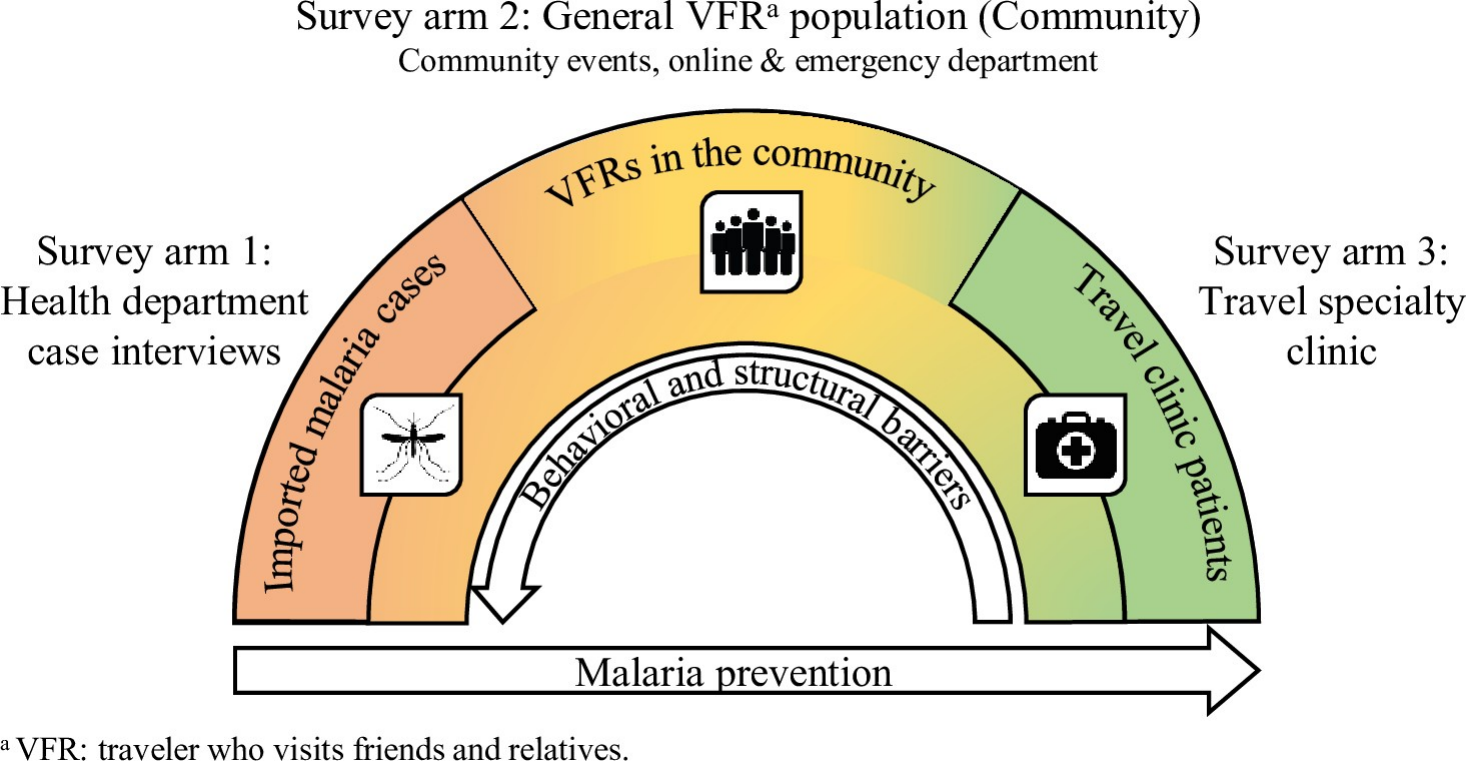
Design of a cross-sectional, multi-setting survey of malaria prevention knowledge, attitudes, and practices among US travelers to sub-Saharan Africa. Participants were surveyed in three study arms. The hypothesized relational directionality of barriers and malaria prevention are shown relative to these three groups, where imported malaria cases were hypothesized to be least effective at preventing malaria and are impacted most strongly by behavioral and structural barriers to malaria prevention.

For this cross-sectional survey, participants were recruited into one of three study arms: 1) imported malaria cases reported to a state health department, 2) the general population of VFR travelers originally from SSA, also referred to as “the community,” and 3) patients (both VFRs and non-VFRs) attending a travel health specialty clinic with upcoming trips to destinations in SSA (Fig 1).

### Eligibility and recruitment

All cases of malaria diagnosed and reported to the Minnesota Department of Health (MDH, St. Paul, Minnesota, USA) were reviewed for eligibility and inclusion in the imported malaria case arm of the study. Consenting US residents diagnosed with incident travel-associated malaria from January 1, 2016, through December 31, 2018, in Minnesota and reported to the state health department, and who traveled for any reason to SSA, were eligible for survey participation. When the case patient was under 18 years of age, only a parent or guardian proxy was eligible to respond on behalf of his or her child. Resettling immigrants, permanent residents of an African nation visiting Minnesota, or people surveyed after April 1, 2019, as a component of ongoing case investigation were ineligible. For the present article, only cases whose reported reason for travel was to visit friends and relatives were included in the analyses.

The VFR general population (community) arm of the study consisted of surveys of past or prospective VFR travelers conducted at community events, online, and at a local emergency department (ED). Survey responses in the community arm were solicited from September 1, 2017, to April 1, 2019. For surveys of the community, eligible VFR travelers were defined as US-resident first- or second-generation immigrants from SSA; 18 years of age or older who had heard of malaria; capable of communicating in English, Amharic, French, or Somali; who either returned from travel to a country in the past 10 years, or plans to travel to a country in the next one year in SSA that is considered endemic for malaria [29]. Community events were public cultural events and occurred within the geographic area in Minnesota where the greatest numbers and proportions of African immigrants live [30]. At the events, study team personnel invited a convenience sample of attendees to participate in the survey. Members of a Community Advisory Board (CAB) [28] also disseminated paper surveys through their organizational and personal networks at cultural events. Online survey participation was promoted through the CAB and community-based organizations. In addition, patients seeking unplanned care for any reason at the Hennepin County Medical Center Emergency Department (HCMC ED, Minneapolis, Minnesota, USA) were invited to participate in the survey while in the waiting room. In the ED, information on triaged patients, including age, place of birth, and ethnicity, was screened by trained surveyors affiliated with the ED for possible eligibility. Persons who presented to the ED had the additional eligibility requirement of presenting in stable mental and physical status. Surveyors approached potential participants to determine final eligibility and interest in survey participation. Purposive sampling by travel region was conducted to attain approximately equivalent sample size targets of West African travelers and travelers returning from other regions of sub-Saharan Africa.

For the travel health clinic arm of the study, patients preparing for travel to countries in SSA with endemic malaria [29] and seeking pretravel care were enrolled from The HealthPartners Travel and Tropical Medicine Center (St. Paul, Minnesota, USA), a local travel medicine clinic serving patients planning travel to a wide range of global travel destinations. Participants were surveyed over the phone before they received pretravel care and recommendations, and again after returning from their trip abroad. Survey responses, including pre- and post-travel components, were solicited among travel clinic respondents from June 1, 2018, to April 1, 2019. The purposive sampling procedure in this arm sought approximately equivalent numbers of VFRs returning from travel to West Africa and SSA, and non-VFR travelers returning from travel to SSA.

### Recruitment targets

**Fig 2:**
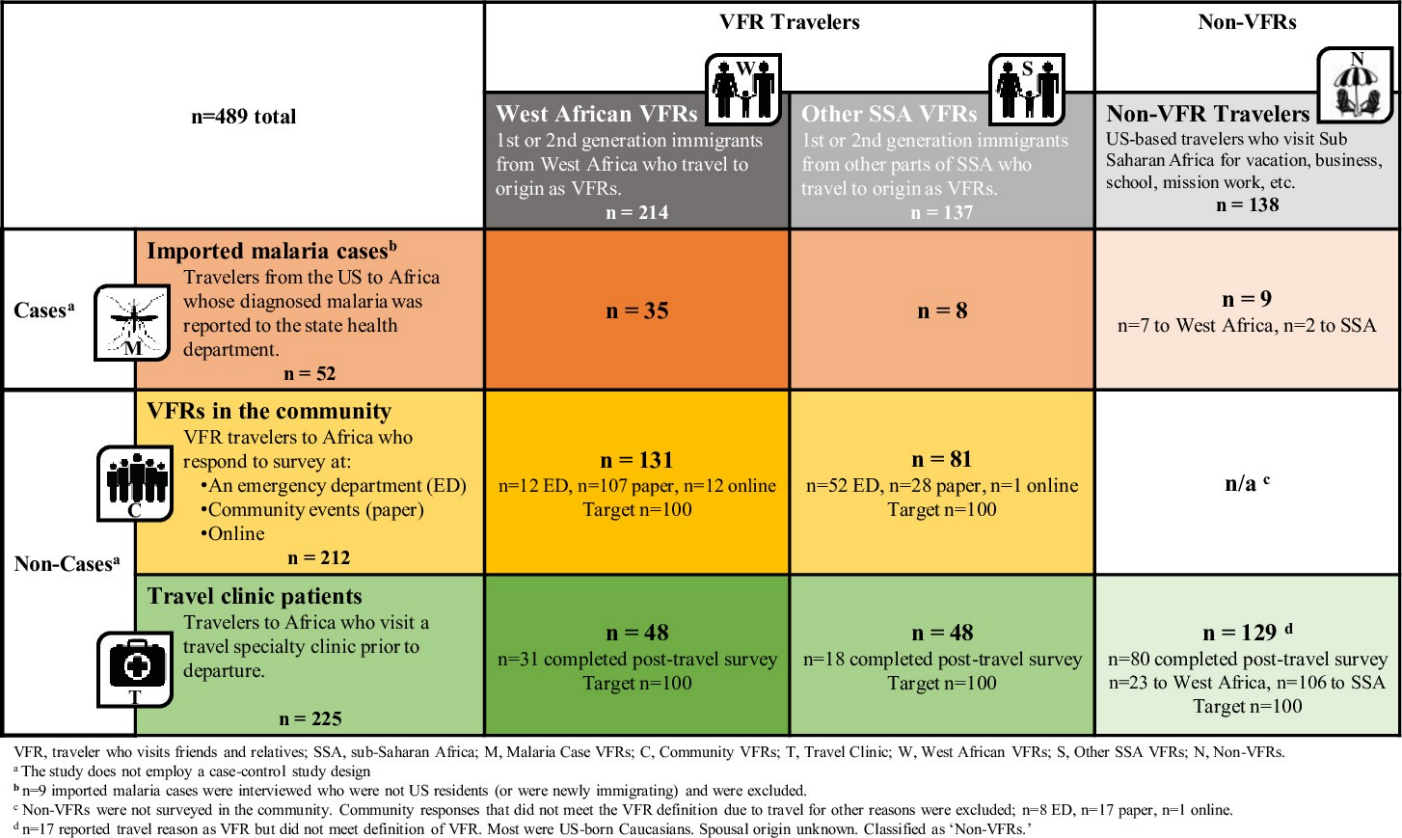
A cross-sectional, multi-setting survey of malaria prevention knowledge, attitudes, and practices among US travelers to sub-Saharan Africa: Participant matrix of target and achieved sample sizes, by survey arm (rows) and traveler population (columns) (n = 489).

A formal sample size calculation was not performed for each arm due to the limited number of eligible respondents in some survey groups, multiple study population subgroups, and lack of reference data in the literature. Within the malaria case arm, participation was limited to reported cases of imported malaria resulting from travel to SSA, and the ability to reach potential respondents through reported phone numbers. The target was to survey 75% of eligible travelers with confirmed malaria where a working phone number was available. The three-pronged approach (ED, events, online) used in the community arm to solicit survey responses was designed to reach a broad VFR traveler population. Target sample size in the community arm was established at 100 West African VFRs and 100 VFRs from other countries of SSA. Finally, a target of more than 100 responses was established for each of the three groups in the travel clinic arm: West African VFRs, other VFRs, and non-VFRs. Fig 2 depicts a matrix of the three survey arms and three travel populations yielding eight subgroups, with the target and final sample size achieved for each subgroup.

### Survey design

Although community-based participatory research has become a more commonly used method in public health studies, it has been used only rarely in travel medicine [28,31] despite often recognized discordance in study team and study population backgrounds. In this study, the survey content and implementation plan were developed through iterative engagement with a CAB of African immigrants in the United States [28]. Additionally, findings from community-based qualitative research conducted by the study team and CAB [13], and a geospatial analysis identifying key study population centers and groups at risk for imported malaria illness [30], provided evidence-based support for decisions on survey content and dissemination.

Survey questions were constructed around malaria KAP, in addition to demographic characteristics. Trip and experiential characteristics were also queried, including destination, frequency of travel, length of stay, travel with children, and past experiences with malaria. Although mosquito coils are not recognized as an effective mosquito avoidance approach, focus groups performed identified that some VFRs use them to avoid bites [13]; a question was included in the survey that addresses their use. A range of categorical, dichotomous, continuous, and Likert scale response fields to prompts were developed around malaria prevention and behavior. Likert scale responses were based on five possible response levels with the value of 1 indicating strongest disagreement, 3 indicating neutrality, and 5 indicating strongest agreement.

A master survey, uniformly replicable across the multiple survey groups, was developed to allow for cross-arm comparison. Survey versions were modified for length and applicability for each setting as noted in Supplemental Materials online.

### VFR and travel region classifications

Survey participants in all three arms were classified as VFR travelers if they met the US Centers for Disease Control and Prevention (CDC) definition for a VFR, “a traveler categorized as a VFR is an immigrant, ethnically and racially distinct from the majority population of the country of residence (a higher-income country), who returns to his or her home country (lower-income country) to visit friends or relatives…” [3] except where the CDC definition includes majority population spouses. Spousal place of birth was not included as a prompt in the surveys. Participants recruited in the malaria case and travel clinic arms of the study were classified as non-VFRs if they did not report VFR travel or if they did not fulfill the requirements of classification as a VFR.

West African travelers were defined as persons traveling to countries within the Global Burden of Disease (GBD) West Africa region [32]. Travelers to other parts of SSA, referred to as “other SSA,” were defined as VFRs traveling to countries within the Southern, Eastern, and Central SSA GBD regions [32], excluding Lesotho because malaria has been eradicated in Lesotho [29]. For survey participants who reported destinations in both West Africa and other sub-Saharan African countries in SSA, VFR participants were classified according to their birth country; non-VFRs were classified as travelers to West Africa if they reported travel to any West African country, regardless of other destinations visited.

### Survey administration

For in-person and online participation, the informed consent process was documented with participants’ signatures or checking a box to indicate consent was granted. In the malaria case arm where participants were surveyed over the phone, the established, approved protocol for the interview of reportable infectious disease cases were followed and included summarizing the reason for the call, explanation of voluntariness, provision of the Tennessen Warning, and written documentation of verbal consent by the surveyor. In the travel clinic arm, the information and consent forms were read to potential participants, verbal consent was documented by the surveyor in the database, and participants received a copy of the information form by mail along with their incentive and educational material. In the malaria case arm, surveys developed for phone calls were not translated; professional telephone interpreters (LanguageLine, Monterey, California, USA) assisted in live, bidirectional interpretation for case surveys when the participant had limited English proficiency. Surveyors attempted to contact cases weekly by telephone up to six times. Surveyors were trained to avoid prompting or validating certain responses while also allowing respondents to elaborate on their experiences as they wished to develop rapport.

For the community and travel clinic arms, surveys, consenting documents, and post-survey informational handouts were professionally translated from English to French, Amharic, and Somali. Global Translator and Interpreter (Minneapolis, Minnesota, USA) translated, back-translated, and certified all translations. At the travel clinic and ED survey sites, surveyors proficient in Somali and French used translated study documents to conduct surveys in person or over the phone, directly in the patient’s preferred language, and later translated the responses back to English. Interpreters in the travel clinic and ED, guided by translated documents, assisted in in-person and telephone survey interpretation when a language-proficient surveyor was unavailable. When surveying the community, paper surveys translated into English, Amharic, French, and Somali were available for participants to self-administer the survey. The online survey was in English only. Participants in the community and travel clinic arms received travel health and malaria-related informational materials upon completion of the survey. Where not prohibited by survey site regulations or Institutional Review Board (IRB) stipulations, participants were eligible for a gift card honorarium for their time.

### Data set integration

A study team member monitored data collection for all arms throughout the survey period and conducted initial and iterative training sessions with surveyors and survey recruiters. REDCap databases (Research Electronic Data Capture, Vanderbilt University, Nashville, Tennessee, USA) were developed and hosted within the Minnesota Department of Health, University of Minnesota, Hennepin County Medical Center, and HealthPartners Institute as approved by IRBs to collect survey responses and monitor recruitment.

All data, including free-response prompts, were reviewed for protected health information by hosting organizations and completely de-identified before integration into a final data set. Variables corresponding to identical or largely identical prompts across the three survey arms were merged in the final data set for comparative analyses.

Subgroups were formed among survey arms to facilitate comparisons of characteristics and outcomes. For analyses limited to participants meeting the VFR definition, a combined group was created that included VFRs in survey arms 2 (community) and 3 (travel clinic), and was termed “non-cases,” to allow for comparison with malaria cases reported to the health department (Fig 2). Within the pool of respondents classified as VFRs, subgroup analyses were also performed to quantify differences by region of travel.

### Statistical analysis and ethical review

Descriptive summary statistics of relevant outcomes and characteristics and comparative analyses stratifying outcomes by survey arm or traveler population were performed. Comparative analyses were performed using Wald’s Chi square statistic (χ^2^) for parametric relationships. Where goodness of fit statistics identified poorly fitting continuous predictors (e.g., duration of travel), predictors were transformed to improve normality and fit; transformations are described where performed. For Likert scale-structured responses, the nonparametric Mann-Whitney U test was used to evaluate pairwise statistical differences due to noncontinuous distribution, plus evidence of skew and non-normality. Odds ratios are presented for comparisons where relevant. The significance level was set for all analyses at α = 0.05. Statistical analyses were performed in SAS version 9.4 (SAS Institute, Cary, North Carolina, USA). Data are visualized in Excel version 2016 (Microsoft, Redmond, Washington, USA).

The following IRBs approved and monitored the survey: Minnesota Department of Health (IRB#15–368), University of Minnesota (STUDY00001189), Hennepin Healthcare Research Institute, formerly Minneapolis Medical Research Foundation of Hennepin County Medical Center (HSR#17–4350), and HealthPartners Institute (IRB0#A14-011). The study was funded by a cooperative agreement from the Centers for Disease Control and Prevention (CDC) (CK000357-01). CDC did not engage in data collection; human subjects review at CDC was not solicited.

## Results

### Population description

A total of 351 VFRs and 138 non-VFR travelers participated across the three survey groups (Fig 2). Among the 351 VFRs, 412 destinations were reported, encompassing 31 countries. The most common destinations in West Africa were Liberia (55; 15.7% of VFR travelers), Nigeria (43; 12.3%), Togo (41; 11.7%), Ghana (26; 7.4%), and Cameroon (22; 6.3%). Destinations in the “other SSA” region were predominantly in East Africa, including Kenya (50; 14.2%), Ethiopia (42; 12.0%), Somalia (35; 10.0%), Sudan (8; 2.3%); and South Africa (8; 2.3%). The most common destinations among the 23 countries reported by the 138 non-VFR travelers were Tanzania (45; 32.6%), Kenya (36; 26.1%), South Africa (25; 18.1%), Ghana (16; 11.6%), Uganda (12; 8.7%), and Zimbabwe (11; 8.0%).

### Characteristics of VFRs

Average and median trip duration among VFRs was 7.0 and 4.0 weeks respectively (range 0.3–72 weeks) (Table 1). Non-normality positive skew of the mean due to multiple long-duration outliers was observed in each study arm and travel region group. After log transformation, duration of travel among other SSA versus West African VFRs was statistically significantly longer (p<0.001) with no differences observed in trip duration across study arms for VFR travelers. Among VFRs, antimalarial use (p = 0.436) or seeing a health care provider before travel (p = 0.362) were not correlated with trip duration.

**Table 1:** A cross-sectional, multi-setting survey of malaria prevention knowledge, attitudes, and practices among US travelers to sub-Saharan Africa: Demographic characteristics and comparisons of participants stratified by survey arm, travel region, and reason for travel.

Part I: Characteristics	All VFRs		VFRs, stratified by survey arm						VFRs, stratified by travel region				All Non-VFRs	
	Overall n = 351		Malaria Cases (M) n = 43		Community(C) n = 212		Travel Clinic(T) n = 96		West African VFRs (W) n = 214		Other SSA VFRs (S) n = 137		Travel Clinic (N) n = 138	
	$\bar{x}$	**(95% CI)**												
Age, years ^a^	43.3	(41.9–44.8)	45.9	(41.4–50.4)	43.1	(41.2–45.0)	42.8	(40.0–45.6)	44.1	(42.4–45.9)	42.2	(39.7–44.7)	51.9	(48.9–55.0)
Trip duration, weeks ^b^	7.0	(6.1–8.0)	7.6	(4.1–11.6)	7.8	(6.3–9.0)	5.4	(4.4–6.3)	5.8	(4.6–6.9)	9.0	(7.4–10.6)	3.8	(2.1–5.6)
Length of residency in US, years ^c^	15.3	(14.4–16.2)	14.3	(11.1–17.5)	14.2	(13.1–15.3)	18.0	(16.1–19.9)	16.1	(14.9–17.4)	14.2	(12.8–15.6)	30.5	(19.8–41.2)
	**%**	**(95%CI)**												
Male	48.2	(42.9–52.6)	72.1	(58.1–86.1)	44.8	(37.9–51.7)	44.6	(34.2–54.9)	51.2	(44.3–58.1)	43.6	(35.1–52.1)	42.5	(33.8–51.2)
Education = grade school	9.5	(6.3–12.7)	11.1	(0.3–21.9)	11.5	(7.0–16.0)	4.4	(0.1–8.7)	4.1	(1.3–6.9)	17.4	(10.9–24.0)	0.8	(0.0–2.3)
Education > high school	68.5	(63.4–73.6)	63.9	(47.4–80.4)	65.5	(58.9–72.1)	76.9	(68.1–85.7)	83.6	(78.3–88.8)	46.2	(37.6–54.8)	95.3	(91.6–99.0)
Foreign-born	95.6	(93.9–98.0)	97.7	(93.0–100)	96.2	(93.6–98.8)	94.6	(89.8–99.3)	94.8	(91.8–97.8)	97.7	(95.3–100)	10.3	(4.9–15.7)
Has had malaria before	67.9	(62.8–72.9)	61.9	(46.6–77.2)	75.2	(69.2–81.2)	53.9	(43.4–64.5)	79.5	(73.9–85.1)	50.4	(41.8–59.0)	3.9	(0.5–7.4)
Has a primary care provider ^d^	88.3	(83.6–93.1)	80.8	(64.5–97.0)	82.5	(72.9–92.2)	94.5	(89.7–99.3)	86.1	(78.3–93.9)	90.0	(84.2–96.0)	88.3	(82.6–93.9)
Destination W. Africa	^e^ 61.0	--	81.4	(69.3–93.5)	^e^ 61.8	--	^e^ 50.0	--	^e^ 100.0	--	^e^ 0.0	--	17.8	(11.1–24.5)
5 or more trips	24.2	(19.5–28.9)	44.0	(24.1–63.9)	20.4	(14.8–26.0)	27.2	(17.9–36.4)	29.4	(22.8–36.0)	16.8	(10.3–23.3)	24.4	(16.8–32.0)
Number of trips	**%**													
Will be first / 1	47.5	--	28.0	--	46.8	--	54.3	--	38.5	--	60.3	--	64.6	--
2 to 4	28.3	--	28.0	--	32.8	--	18.5	--	32.1	--	22.9	--	11.0	--
5 or more	24.2	--	44.0	--	20.4	--	27.2	--	29.4	--	16.8	--	24.4	--
**Part II: Comparisons**	**M *vs*. C**	**M *vs*. T**	**C *vs*. T**	**W *vs*. S**	**T *vs*. N**
	**Pairwise Wald χ**^**2**^ **(*df = 1*) p-value**				
Age, years ^a^	0.222	0.235	0.868	0.197	*<0.001
Trip duration, weeks ^b^	0.873	0.288	0.092	*<0.001	*<0.001
Length of residency in US, years ^c^	0.960	0.058	*0.001	*0.044	*0.001
Male	*0.002	*0.004	0.967	0.172	0.763
Education = grade school	0.947	0.175	0.062	*<0.001	0.117
Education > high school	0.852	0.138	0.052	*<0.001	*<0.001
Foreign-born	0.642	0.428	0.513	0.184	*<0.001
Has had malaria before	0.080	0.391	*<0.001	*<0.001	*<0.001
Has a primary care provider ^d^	0.843	*0.038	*0.023	0.406	0.123
Destination W. Africa	--	--	--	--	--
5 or more trips	*0.008	0.108	0.199	*0.011	0.644
Number of trips	**Pairwise Wald χ**^**2**^ **(df = 2) p-value**				
Will be first / 1	*0.034	0.075	*0.040	*0.001			0.209
2 to 4							
5 or more							

VFR, traveler who visits friends and relatives; SSA, sub-Saharan Africa; M, Malaria Case VFRs; C, Community VFRs; T, Travel Clinic VFRs; W, West African VFRs; S, Other SSA VFRs; N, Non-VFRs at the Travel Clinic; $\bar{x}$, mean; %, percent.

Column colors correspond to the groups described and depicted in Fig 2.

^a^ n = 3 pediatric cases excluded from calculation of mean age for comparison across groups. Mean age in years including pediatric cases was 43.3 (38.3–48.6).

^b^ Pairwise comparisons use log-transformed trip duration due to non-normality positive skew of the mean.

^c^ Foreign-born respondents only.

^d^ Paper and online versions of the community survey omitted this prompt therefore community survey arm is ED respondents exclusively.

^e^ Noninformative; sampling protocol-determined outcome.

* Alpha (α) = 0.05; p-value is statistically significant.

VFRs at the travel clinic were US residents longer than VFRs surveyed in the community (18.0 years versus 14.2 years, p = 0.001). Having a primary care provider was reported among 88.3% (159 of 180) of VFRs; travel clinic VFRs (86 of 91; 94.5%) were more likely than community VFRs (surveyed in the ED only) (52 of 63; 82.5%; p = 0.023) or travelers with malaria (21 of 26; 80.8%; p = 0.038) to have a primary care provider.

VFRs who reported taking an antimalarial on their last trip (164 of 276; 59.4%) were statistically significantly more concerned about malaria (mean [$\bar{x}$] = 3.8, median [$\tilde{x}$] = 4) than those who did not take an antimalarial ($\bar{x}$ = 3.1, $\tilde{x}$ = 3; p = 0.001). Using insect repellent (131 of 211; 62.1%) was also associated with greater concern for malaria ($\bar{x}$ = 3.7, $\tilde{x}$ = 4) compared to VFRs who did not use repellent ($\bar{x}$ = 3.1, $\tilde{x}$ = 3; p = 0.010). Taking an antimalarial or using repellent was not statistically associated with perception of malaria deadliness among VFRs.

VFRs overwhelmingly recognized that malaria is a preventable illness (323 of 344; 93.9%), but some differences across groups were observed (Table 2): VFRs at the travel clinic (94 of 95; 98.9%) were more likely to perceive malaria as preventable than VFRs in the community (190 of 207; 91.8%; p = 0.040). Other SSA VFRs (121 of 134; 90.3%) were less likely than West African VFRs to perceive malaria as preventable (202 of 210; 96.2%; p = 0.031).

**Table 2:** A cross-sectional, multi-setting survey of malaria prevention knowledge, attitudes, and practices among US travelers to sub-Saharan Africa: Malaria knowledge, attitudes and practices among travelers visiting friends and relatives, across study arms and regional traveler populations.

Part I: Characteristics	All VFRs		VFRs, stratified by survey arm						VFRs, stratified by travel region			
	Overall n = 351		Malaria Cases (M) n = 43		Community (C) n = 212		Travel Clinic (T) n = 96		West African VFRs (W) n = 214		Other SSA VFRs (S) n = 137	
** **	$\tilde{x}$	$\bar{x}$ **(95%CI)**	** **	** **	** **	** **	** **	** **	** **	** **	** **	** **
Concern about malaria (Likert, 1–5, low-high)	4	3.6 (3.4–3.7)	1	2.5 (1.9–3.1)	4	3.8 (3.6–4.0)	4	3.5 (3.2–3.8)	4	3.7 (3.5–3.9)	3	3.3 (3.0–3.6)
Malaria is deadly (Likert, 1–5, disagree-agree)	5	4.5 (4.4–4.6)	5	4.0 (3.6–4.5)	5	4.6 (4.5–4.7)	5	4.5 (4.3–4.6)	5	4.6 (4.4–4.7)	5	4.4 (4.3–4.6)
	**%**	**(95%CI)**										
Malaria is preventable	93.9	(91.4–96.4)	92.9	(84.7–100)	91.8	(88.0–95.6)	98.9	(96.7–100)	96.1	(93.6–98.8)	90.2	(85.2–95.4)
Listed incorrect method of preventing malaria	39.9	(34.3–45.4)	57.5	(41.5–73.5)	36.2	(29.0–43.4)	39.1	(29.0–49.3)	33.5	(26.5–40.6)	48.5	(39.8–57.2)
Listed incorrect method of contracting malaria	19.5	(15.2–23.7)	16.3	(4.8–27.8)	16.5	(11.3–21.7)	27.1	(18.0–36.1)	14.2	(9.4–19.0)	27.4	(19.8–35.0)
Had malaria on a previous trip ^a^	11.1	(6.9–15.2)	^e^100	--	12.5	(7.3–17.7)	7.6	(1.0–14.1)	14.4	(8.3–20.5)	6.4	(1.3–11.4)
Saw health care provider before last trip ^b^	66.8	(60.4–73.2)	41.9	(26.5–57.2)	73.2	(66.4–80.0)	^e^100	--	69.9	(62.3–77.5)	60.3	(48.4–72.2)
On most recent past trip…												
Took an antimalarial	59.4	(53.6–65.2)	31.7	(16.8–46.6)	61.9	(54.5–69.3)	^c^70.1	(58.9–81.4)	66.7	(59.6–73.7)	47.1	(37.2–56.9)
Picked where to stay to avoid mosquitos	57.2	(50.2–64.2)	19.2	(3.0–35.5)	63.1	(55.7–70.5)	^d^	--	62.0	(53.5–70.5)	47.7	(35.2–60.2)
Educated oneself about malaria	57.7	(50.7–64.7)	23.1	(5.7–40.4)	63.1	(55.7–70.5)	^d^	--	60.5	(51.9–69.0)	52.3	(39.8–64.8)
Used mosquito repellent	62.1	(55.5–68.7)	46.5	(31.0–62.0)	66.1	(58.8–73.3)	^d^	--	67.1	(59.3–74.9)	51.5	(39.3–63.7)
Stayed indoors when mosquitos were out	55.2	(48.1–62.2)	26.9	(8.7–45.2)	59.5	(52.0–67.0)	^d^	--	55.0	(46.3–63.7)	55.4	(43.0–67.8)
Wore long clothing	58.2	(51.2–65.2)	26.9	(8.7–45.2)	63.1	(55.7–70.5)	^d^	--	55.0	(46.3–63.7)	64.6	(52.7–76.6)
Used bed nets	59.7	(53.0–66.4)	44.2	(28.7–59.7)	63.7	(56.3–71.0)	^d^	--	57.3	(49.1–65.5)	64.7	(53.1–76.4)
Used mosquito coil	28.4	(22.0–34.7)	19.2	(3.0–35.5)	29.8	(22.8–36.7)	^d^	--	30.2	(22.2–38.3)	24.6	(13.9–35.4)
**Part II: Comparisons**	**M *vs*. C**	**M *vs*. T**	**C *vs*. T**	**W *vs*. S**
	**Mann-Whitney U two-sample test p-value**			
Concern about malaria (Likert, 1–5, low-high)	*<0.001	*0.002	0.174	*0.014
Malaria is deadly (Likert, 1–5, disagree-agree)	*0.003	0.170	*0.035	0.354
	**Pairwise Wald χ**^**2**^ **(df = 1) p-value**			
Malaria is preventable	0.816	0.091	*0.04	*0.031
Listed incorrect method of preventing malaria	*0.015	0.053	0.639	*0.009
Listed incorrect method of contracting malaria	0.972	0.171	*0.034	*0.003
Had malaria on a previous trip ^a^	--	--	0.288	0.065
Saw health care provider before last trip ^b^	*<0.001	--	--	0.166
On most recent past trip…				
Took an antimalarial	*0.001	*<0.001	0.235	*0.002
Picked where to stay to avoid mosquitos	*<0.001	--	--	0.058
Educated oneself about malaria	*<0.001	--	--	0.278
Used mosquito repellent	*0.020	--	--	*0.029
Stayed indoors when mosquitos were out	*0.003	--	--	0.9635
Wore long clothing	*0.001	--	--	0.203
Used bed nets	*0.022	--	--	0.309
Used mosquito coil	0.273	--	--	0.413

VFR, traveler who visits friends and relatives; SSA, sub-Saharan Africa; M, Malaria Case VFRs; C, Community VFRs; T, Travel Clinic VFRs; W, West African VFRs; S, Other SSA VFRs; $\tilde{x}$, median; $\bar{x}$, mean; %, percent.

Column colors correspond to the groups described and depicted in Fig 2.

^a^ Overall, W, S, and their pairwise OR exclude malaria case participants

^b^ Overall, W, S, and their pairwise OR exclude travel clinic participants

^c^ Reflects responses from the pretravel survey regarding most recent past trip prior to scheduling a pretravel visit at the travel clinic.

^d^ Travel clinic VFR respondents were not surveyed regarding malaria prevention techniques on trips prior to scheduling the pretravel visit besides antimalarial use. Post-travel results on malaria prevention for the travel clinic VFR group are reported in Table 3.

^e^ Noninformative; sampling protocol-determined outcome

* Alpha (α) = 0.05; p-value is statistically significant.

### Characteristics of malaria cases in VFRs

Slightly fewer than half (163 of 338; 48.2%) of all VFRs were male; travelers with malaria were disproportionately male (31 of 43; 72.1%) compared to the community (91 of 203; 44.8%; p = 0.002) or travel clinic (41 of 92; 44.6%; p = 0.004) VFR respondents. Malaria case VFRs (11 of 25; 44.0% took 5+ trips) had 2.7 times greater odds of having taken five or more trips than non-case VFRs (66 of 293; 22.5% took 5+ trips) (odds ratio [OR] = 2.70; 95% confidence interval [95% CI] = 1.17–6.24; p = 0.020). Five or more trips to SSA while residing in the US were reported by 44.0% of malaria cases (11 of 25); fewer case respondents took two to four trips to SSA (7 of 25; 28.0%) or became ill on their first trip to SSA (7 of 25; 28.0%).

Malaria case VFRs ($\tilde{x}$ = 1) were statistically significantly less concerned about malaria before travel than the community ($\tilde{x}$ = 4; p<0.001) or travel clinic VFRs ($\tilde{x}$ = 4; p = 0.002). A significant difference in distribution of scores was observed between malaria case VFRs and community VFRs (p = 0.003), and between community VFRs and travel clinic VFRs (p = 0.035) (Table 2). A statistically significantly larger proportion of malaria case VFRs (23 of 40; 57.5%) listed an incorrect prevention approach in comparison to VFRs in the community (63 of 174; 36.2%; p = 0.015).

Overall, non-case VFRs (151 of 235; 64.3% used antimalarial) had 3.9 times greater odds of using an antimalarial than diagnosed malaria case VFRs (13 of 41; 31.7% used antimalarial) (OR = 3.87; 95% CI = 1.90–7.87; p<0.001). Nearly three-quarters (123 of 168; 73.2%) of community VFRs saw a health care provider before their last trip, whereas 41.9% (18 of 43) of malaria cases saw a provider (p<0.001). Community VFRs were more likely than travelers with malaria to use preventive behaviors (Table 2), including picking where to stay to avoid mosquitoes, educating oneself on the risk of malaria during travel, using mosquito repellent, staying indoors when mosquitoes were biting, wearing long clothing, and using bed nets.

### Efficacy of implementing preventive methods in VFRs and non-VFRs: Travel clinic

VFRs traveled statistically significantly longer (mean 5.4 weeks; range 1–39 weeks) than non-VFRs (mean 3.8 weeks; range 0.3–104 weeks) in the travel clinic (p<0.001). There was no difference in the number of prior trips between non-VFRs and VFRs.

**Fig 3:**
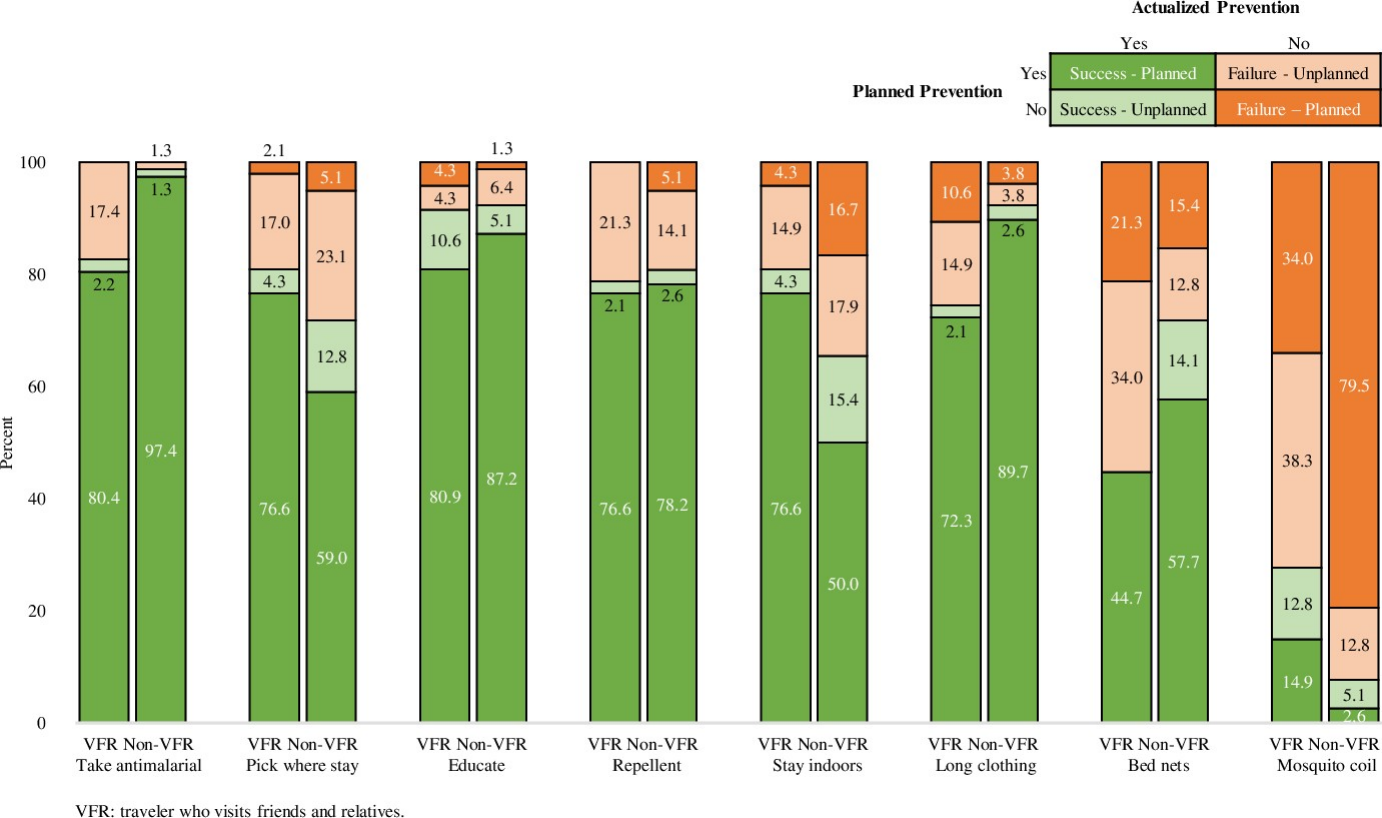
A cross-sectional, multi-setting survey of malaria prevention knowledge, attitudes, and practices among US travelers to sub-Saharan Africa: Planned and actual malaria prevention outcome matrix among travelers visiting friends and relatives (VFRs) and non-VFRs surveyed at the travel clinic both before and after travel.

**Fig 4:**
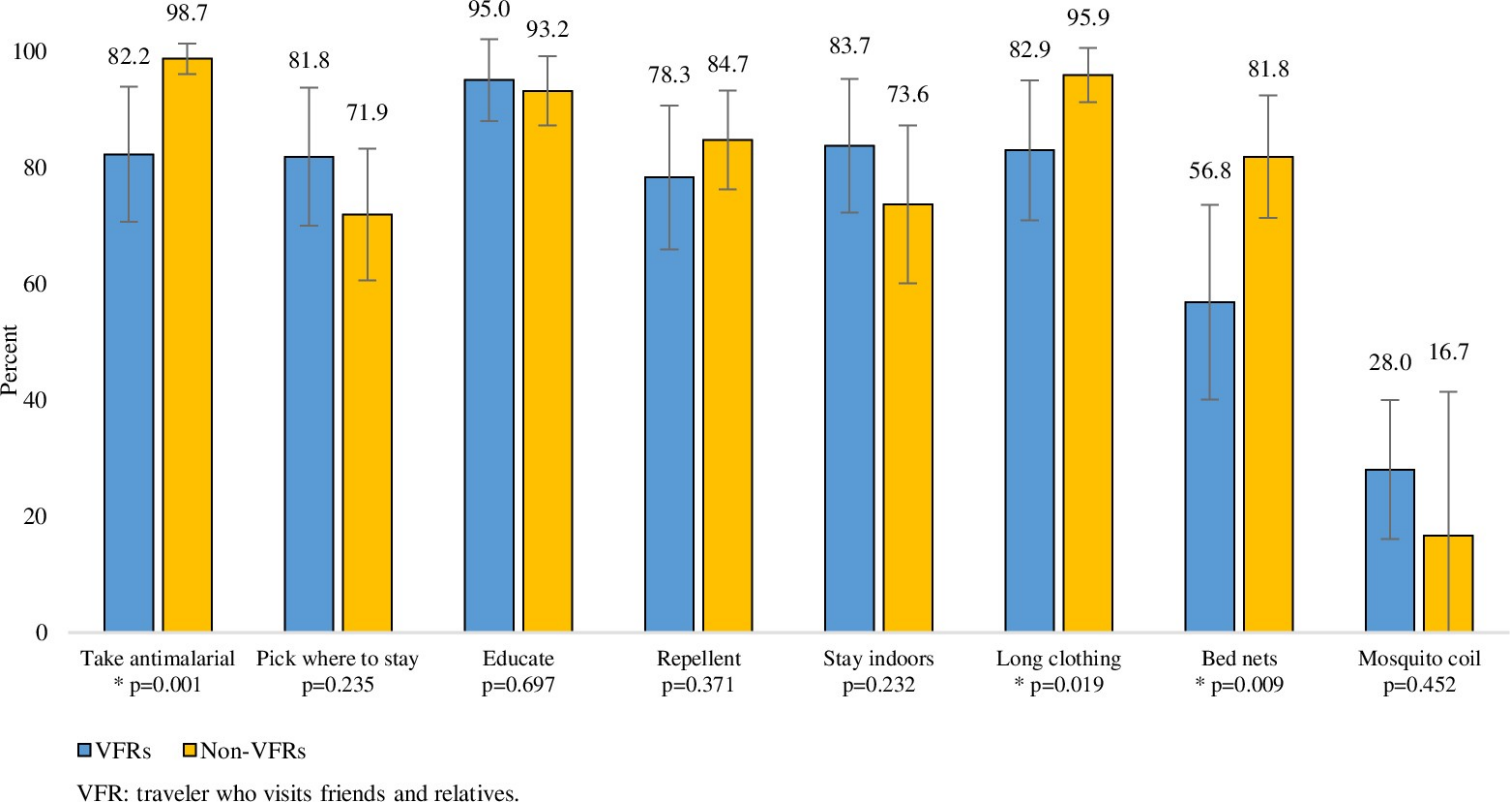
A cross-sectional, multi-setting survey of malaria prevention knowledge, attitudes, and practices among US travelers to sub-Saharan Africa: Adherence to malaria prevention reported after travel among travelers visiting friends and relatives (VFRs) and non-VFRs who reported planning to use the prevention approach before travel.

Before their trip, travel clinic VFRs were more likely than non-VFRs to plan to use certain malaria prevention approaches including: picking where they stayed to avoid mosquitoes ([90 of 96; 93.8%] versus [108 of 129; 83.7%]; p = 0.011), staying indoors when mosquitoes are out ([84 of 96; 87.5%] versus [88 of 129; 68.2%]; p = 0.001), and using mosquito coils ([44 of 96; 45.8%] versus [22 of 129; 17.1%]; p<0.001) (Table 3). Figs 3 and 4 demonstrate the proportion of VFRs and non-VFRs who were successful in following through with their planned malaria prevention. Compared to non-VFR travelers, VFRs were less successful in taking antimalarials ([75 of 76; 98.7%] versus [37 of 45; 82.2%]; p = 0.001), wearing long clothing ([70 of 73; 95.9%] versus [34 of 41; 82.9%]; p = 0.019), and using bed nets ([45 of 55; 81.8%] versus [21 of 37; 56.8%]; p = 0.009).

**Table 3:** A cross-sectional, multi-setting survey of malaria prevention knowledge, attitudes, and practices among US travelers to sub-Saharan Africa: Inter- and intra-population differences among travelers visiting friends and relatives (VFRs) and other types of travelers to similar destinations (non-VFRs), by survey arm and traveler population.

Characteristics	Survey arm: Community		Survey arm: Travel Clinic				Comparisons	
							Intra-VFR	Inter-Pop
	Community VFRs (C) n = 212		Travel Clinic VFRs (T) n = 96		Non-VFRs (N) n = 129		C *vs*. T p-value	T *vs*. N p-value
**Malaria Knowledge & Attitudes**	$\tilde{x}$	$\bar{x}$ **(95% CI)**					**Mann-Whitney U**	
Concern about malaria (Likert, 1–5, low-high)	4	3.8 (3.6–4.0)	4	3.5 (3.2–3.8)	3	2.7 (2.5–2.9)	0.174	*<0.001
Malaria is deadly (Likert, 1–5, disagree-agree)	5	4.6 (4.5–4.7)	5	4.5 (4.3–4.6)	5	4.5 (4.4–4.7)	*0.035	0.196
	**%**	**(95% CI)**					**Wald χ2 (*df = 1*)**	
Malaria is preventable	91.8	(88.0–95.6)	98.9	(96.7–100)	96.9	(93.9–99.9)	*0.040	0.328
Listed incorrect method of preventing malaria	36.2	(29.0–43.4)	39.1	(29.0–49.3)	27.6	(19.7–35.4)	0.639	0.072
Listed incorrect method of contracting malaria	16.5	(11.3–21.7)	27.1	(18.0–36.1)	16.3	(9.8–22.7)	*0.034	0.051
Had malaria on a previous trip	12.5	(7.3–17.7)	7.6	(1.0–14.1)	3.6	(0.0–8.6)	0.288	0.354
**Planned** ^a^ **& Actualized** ^b^ **Malaria Prevention**	**%**	**(95% CI)**					**Wald χ2 (*df = 1*)**	
Will take antimalarial	94.6	(87.0–100)	97.8	(94.8–100)	99.2	(97.6–100)	0.349	0.416
Did take antimalarial	61.9	(54.5–69.3)	83.0	(71.8–94.1)	98.7	(96.2–100)	*0.009	*0.011
Will pick where to stay to avoid mosquitos	88.9	(78.1–99.7)	93.8	(88.8–98.7)	83.7	(77.3–90.2)	0.353	*0.027
Did pick where to stay to avoid mosquitos	63.1	(55.7–70.5)	80.9	(69.2–92.5)	71.8	(61.6–82.0)	*0.025	0.259
Will educate oneself about malaria	91.7	(82.2–100)	91.7	(86.0–97.3)	95.3	(91.7–99.0)	1.000	0.264
Did educate oneself about malaria	63.1	(55.7–70.5)	91.5	(83.2–99.8)	92.3	(86.3–98.4)	*0.001	0.870
Will use mosquito repellent	83.3	(70.5–96.1)	91.7	(86.0–97.3)	95.3	(91.7–99.0)	0.174	0.264
Did use mosquito repellent	66.1	(58.8–73.3)	78.7	(66.6–90.9)	80.8	(71.8–89.7)	0.102	0.782
Will stay indoors when mosquitos are out	73.3	(70.5–96.1)	87.5	(80.8–94.2)	68.2	(60.1–76.4)	0.536	*0.001
Did stay indoors when mosquitos were out	59.5	(52.0–67.0)	80.9	(69.2–92.5)	66.2	(55.4–77.0)	*0.009	0.083
Will wear long clothing	77.8	(63.5–92.0)	88.5	(82.1–95.0)	94.6	(90.6–98.5)	0.123	0.106
Did wear long clothing	63.1	(55.7–70.5)	74.5	(61.5–87.4)	92.3	(86.3–98.4)	0.150	*0.009
Will use bed nets	91.7	(82.2–100)	79.2	(70.9–87.4)	69.8	(61.7–77.8)	0.104	0.115
Did use bed nets	63.7	(56.3–71.0)	44.7	(29.9–59.4)	71.8	(61.6–82.0)	*0.020	*0.003
Will use mosquito coil	63.9	(47.4–80.4)	45.8	(35.7–56.0)	17.1	(10.5–23.6)	0.067	*<0.001
Did use mosquito coil	29.8	(22.8–36.7)	24.4	(11.4–37.5)	7.7	(1.6–13.7)	0.484	*0.013

VFR, traveler who visits friends and relatives; C, Community VFRs; T, Travel Clinic VFRs; N, Non-VFRs at the Travel Clinic; $\tilde{x}$, median; $\bar{x}$, mean; %, percent.

Column colors correspond to the groups described and depicted in Fig 2.

^a^ Community VFR group includes only respondents who have not yet traveled, reporting planned malaria prevention for first trip (n = 37). Travel clinic VFR (n = 96) and Non-VFR (n = 129) groups include all pretravel respondents.

^b^ Community VFR group includes only respondents who had traveled, reporting malaria prevention used on last trip (n = 168). Travel clinic VFR (n = 49) and Non-VFR (n = 80) groups include only travelers who completed the post-travel survey

* Alpha (α) = 0.05; p-value is statistically significant.

## Discussion

While immigrant health outcomes are often studied in relation to assimilation to their new home country [33], established immigrants represent a mobile population with intact linkages to other parts of the world [15]. An international trip to visit friends and relatives requires considerable planning and expense [13]; ensuring preparation for prevention of endemic diseases at the destination is critical. For health care providers, asking about upcoming travel could be a useful screening tool for providing appropriate preventive health care to this vulnerable population.

This is the first epidemiologically robust evaluation of malaria KAP and barriers to prevention with evidence to challenge many long-held beliefs around VFR behavior. In the travel clinic, non-VFRs reported statistically significantly lower concern about acquiring malaria than VFRs, while differences in level of concern were not observed between VFRs surveyed in the travel clinic and VFRs community. This challenges common assumptions that VFRs are less worried about malaria than other types of travelers [14, 15, 24, 33, 34]. Additionally, no differences in knowledge about malaria prevention and transmission were observed between VFRs and non-VFRs, yet it is well-documented that success among VFRs in realizing malaria prevention is lower than that of non-VFRs [8–11]. In our study, the VFR group hypothesized to be the most proactive and well-connected to preventive care (VFRs at the travel clinic) were still less successful at malaria prevention than non-VFRs attending the same travel clinic. Similar differences have been noted in immigrants in other areas of preventive health, such as decreased rates of cancer screening relative to white, native-born US citizens [21]; this holds true even after controlling for socioeconomic and insurance status [35].

### The heterogeneity of the VFR population

Although commonly described as a single population, VFR traveler subgroups may vary in their adherence to malaria prevention and in their interactions with health care systems [10,12,27]. Studies of VFRs residing in other countries and engaging with the health care systems in those countries have shown that differences in malaria prevention behaviors are broad, and the heterogeneity of experiences among immigrant VFRs should not be understated [36–38].

Our results demonstrated unique differences among VFRs from SSA, even among those residing in one area of the United States. Compared to West African VFRs, East African VFRs who made up most of the “other SSA” VFR subgroup, travel for longer duration, had a shorter length of United States residency, a lower likelihood of a postsecondary education, and have taken fewer trips as VFRs. As these newer arrivals to the United States become more settled, we expect that East African immigrants may become an emerging traveler population to destinations with malaria. Future interventions developed in anticipation of increased travel within this group that account for unique needs such as longer travel duration and lower education may be more impactful within this group.

When comparing VFRs by location of pretravel care, VFRs at the travel clinic had a greater level of awareness that malaria can be prevented as compared to other VFRs surveyed in the community who may have had no pretravel health care or visited only a primary care provider. Travel clinic VFRs were also more likely to report having a primary care provider than malaria cases or VFRs surveyed in the community, suggesting travel clinic VFRs may reflect a sub-population more familiar with preventive health, better linked to, or better able to navigate the health care system. However, the findings for the community may be biased since emergency department users may be less likely to have a primary care provider. Maintaining an identified primary care provider has been shown to be protective for other preventable health conditions in US immigrant populations [22,35], while other factors like socioeconomic status and education level were not protective [21,35].

Although reported antimalarial use among community VFRs was greater [16] or similar [39] to reports elsewhere in the literature, it was still statistically significantly less than antimalarial use among travel clinic VFRs. Travel clinic VFRs were also more likely to report mosquito avoidance approaches. Considering that travel clinic VFRs are better linked to preventive care than community VFRs, this tendency for greater malaria prevention may be confounded by increased preventive behaviors generally. Interventions that integrate community-based approaches outside of traditional health care touch points may be necessary to reach VFRs less linked to preventive care.

One finding identified across all VFR subpopulations in this convenience sample of travelers from a variety of settings was that while malaria knowledge may not have been strongly associated with malaria prevention, attitudes and risk perception for malaria did correlate with preventive behavior. Taking an antimalarial or using insect repellent was associated with statistically significantly higher concern for malaria during travel. There are also potential structural barriers more likely to impact VFR travelers than others taking trips abroad. VFRs consistently report trips of long duration; in preparation for such travel, VFRs may face barriers in obtaining sufficient antimalarial medication to cover the entire length of their visits, especially when Medicaid or private insurance plans in the United States limit antimalarial dispensing to a 34-day supply and may not permit vacation overrides [40].

### Differences in the characteristics of malaria cases and other VFR travelers

By comparing the characteristics of post-travel malaria cases with the characteristics of a broader representation of VFR travelers from the same area, we can begin to form hypotheses about some of the behavioral and demographic risk factors related to prevention failure. Males were greatly overrepresented among surveyed malaria cases compared to VFRs surveyed in the community and travel clinic groups. This is consistent with international surveillance reports for imported malaria to nonendemic, high income countries [41]. In other US immigrant populations, males have also been shown to have lower health literacy than females [18], and to exhibit more risk-taking behaviors [42].

It is possible that multiple factors contribute to failure of malaria prevention in VFR travelers. Although having largely comparable knowledge about malaria, diagnosed case VFRs were more likely than other VFRs to list incorrect or non-existent methods of preventing malaria—most commonly, use of malaria vaccine. This suggests that misconceptions around being immunized against malaria could provide some VFRs with a false sense of protection, inhibiting their use of other (more effective) preventive measures.

While participants surveyed in other settings were excluded if they had not heard of malaria before, this was not an exclusion criterion for malaria cases under the presumption that due to their diagnosis, cases had some knowledge of malaria at the time of the survey. Malaria cases may have been included that had not heard of malaria prior to becoming ill and thus may have been more likely to report incorrect methods of preventing malaria. Based upon the infrequency that this criterion excluded participants in other settings, the anticipated effect of this potential bias in baseline knowledge is hypothesized to be negligible or minimal. Overall, neither listing an incorrect prevention approach or method of contracting malaria were statistically correlated with decreased antimalarial use.

**Fig 5:**
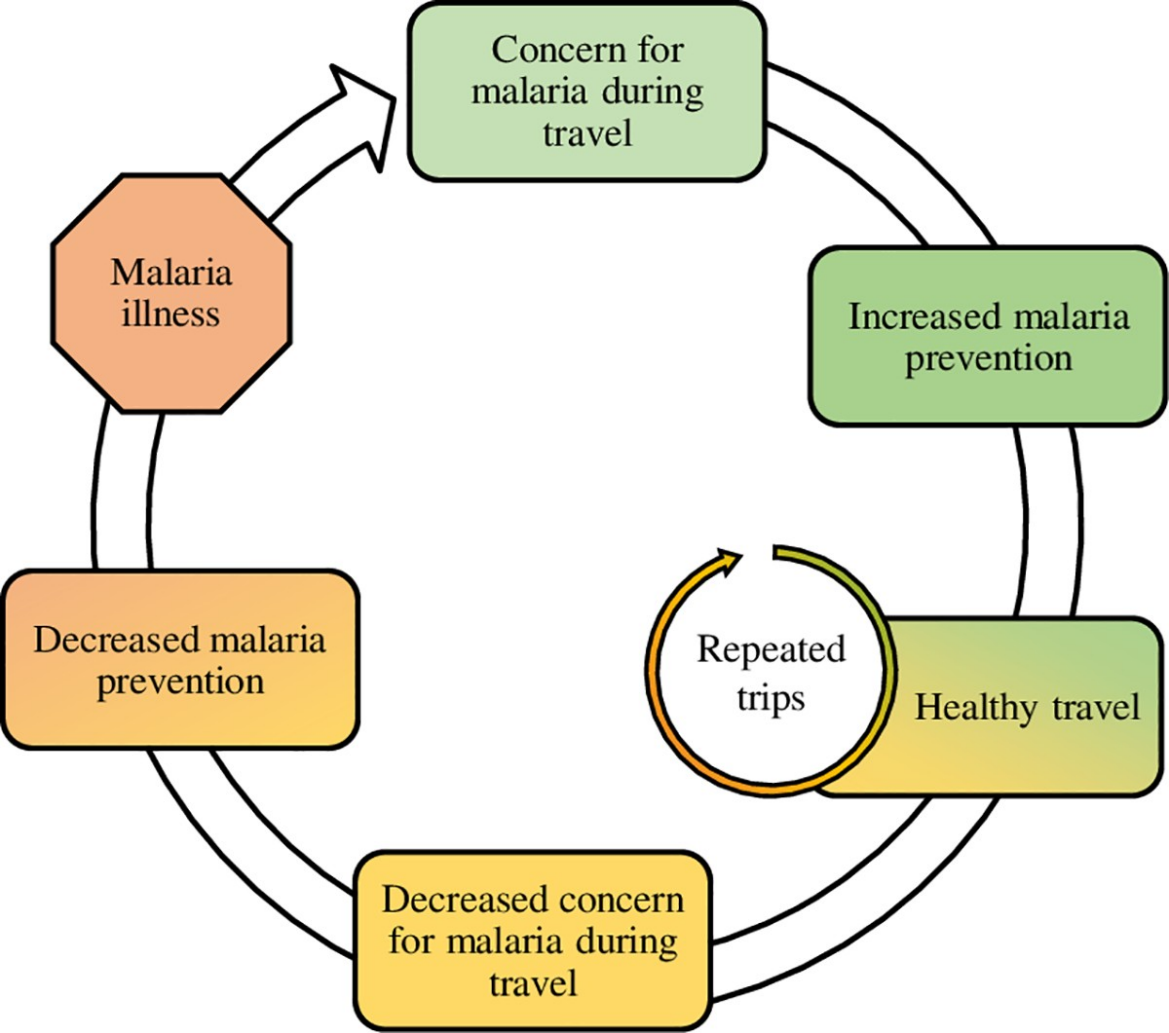
A cross-sectional, multi-setting survey of malaria prevention knowledge, attitudes, and practices among US travelers to sub-Saharan Africa: Conceptual health behavior framework with hypothesized reduction in risk perception and use of malaria prevention upon repeated trips of travelers visiting friends and relatives.

Travelers who contracted malaria during their most recent trip appeared to be more frequent travelers than other VFR travelers and reported lower concern about malaria before travel. These differences suggest that risk perception and preventive behaviors may wane among VFRs after repeated healthy trips (Fig 5). Repeated healthy travel could thus act as a barrier to malaria prevention among VFRs. Similar patterns of risk perception burnout have been observed in young adults with health conditions such as type 1 diabetes that require continued vigilance and proactive management, leading to poorer disease control over time [43,44].

### Study strengths and limitations

The three-arm approach used to survey VFRs demonstrated differences in stratified subpopulations and reflected a broad range of malaria prevention KAP. However, because of the lack of a known, representative VFR population or reliable census data describing this group, the representativeness of the study population cannot be estimated. The extent to which non-VFRs surveyed in the travel clinic reflect the underlying traveling population to malaria-endemic areas is unknown and was not explored in the study design. Non-VFRs were only surveyed through their engagement with pretravel care; for this reason, malaria prevention may be overestimated in this population. Expanded or targeted community-based studies would be necessary to achieve a more representative sample of the overall traveler population.

Sampling techniques in this study targeted areas with large numbers of immigrants from SSA and may be representative of the Minnesota VFR population. The study may not be generalizable to other African VFR population centers in the United States or internationally. However, findings from focus groups performed with West African VFR travelers both in Minneapolis-St. Paul and New York City [13] do align strongly with the survey findings on behavioral barriers to malaria prevention.

When identifying study participants with documented cases of malaria, we were limited to recruiting those diagnosed in Minnesota, and likely underestimated both persons who became ill and were treated abroad, as well as those who did not seek diagnosis and treatment at a United States hospital upon return from travel. A malaria-like illness on a past trip was self-reported by 11% of VFR travelers in community and travel clinic groups. As many illnesses present with similar symptoms to malaria, this is likely an overestimation of the malaria prevalence in the VFR traveler population, but it suggests that there may be unreported malaria cases in this population, especially if they self-treat their symptoms with antimalarials procured prior to travel or while abroad. During the study period, potentially eligible cases were lost to follow up as many had non-working phone numbers. Systematic and directional bias could affect case responses towards respondents more willing to talk to a state government representative, or towards those with more reliable phone accounts. Age and gender distribution of sampled cases was not statistically significantly different from all reported malaria cases in Minnesota during the same time period.

In all populations, social desirability and correct answer bias could skew results toward the over-reporting of preventive behaviors or affirmative reporting of behaviors used inconsistently. Despite this, the differential between planned and adhered to preventive behaviors should theoretically remained constant and reliable, assuming prevention was overreported equally among travelers who had traveled and those who were planning to travel soon. Attempts to minimize this source of bias were incorporated into survey design, and techniques were used to develop rapport with respondents and pose the questions without prompting a preferred answer.

## Conclusion

As the gold standard for malaria prevention in travelers, correct use of chemoprophylaxis is critical. Consistent with recent literature, this study found that malaria knowledge alone did not translate to successful malaria prevention. Interventions that communicate to VFRs the severity of malaria illness, the importance of pretravel health care, and the necessity for chemoprophylaxis would address barriers identified in this study. Strategies to make intended malaria prevention easier, more affordable, and more accessible, can help improve the likelihood of adherence to recommendations.

Although VFRs at the travel clinic may be better engaged with preventive and pretravel health care than their VFR peers surveyed in the community, specialized pretravel care does not appear to ensure chemoprophylaxis use among VFRs. Raising the level of concern for malaria and communicating its severity while also conveying the high level of effectiveness of malaria prevention when followed properly may be key messages that increase uptake of chemoprophylaxis among VFRs. Improving access to primary care and general preventive health in immigrant populations may have impacts on issues such as malaria and travelers’ health.

Heterogeneity of barriers to malaria prevention exist within VFR groups and across traveler populations. Addressing access to health care and upstream barrier reduction strategies that make intended prevention more achievable, affordable, easier, and resonant among VFRs may improve malaria prevention intervention effectiveness. Future research needs include working to quantify the more impactful behavioral barriers proposed in the literature specifically in the VFR population instead of across traveler populations broadly. The detail of analysis and subgroup analysis presented in this study may serve as a model to be replicated in future work when examining the VFRs’ specific barriers to malaria prevention.

## Supporting information

S1 Checklist STROBE checklist. Checklist of reporting guidelines for observational studies.
(PDF)

S1 Dataset Data set and data dictionary. Data set contains de-identified data for eligible records and an accompanying data dictionary.
(XLSX)

S1 Document Survey material packet. Surveys and consent materials are included for the five survey versions used across the three study arms. Materials are ordered as follows: S2.1) Consent and Survey– 2016 Case Interview—Pilot Instrument; S2.2) Consent and Survey–Final Case Interview; S2.3) Consent and Survey–Emergency Department; S2.4) Consent and Survey–Community Paper and Online; S2.5) Consent and Survey–Travel Clinic.
(PDF)
