# Supplementary material for: Barriers to malaria prevention among immigrant travelers in the United States who visit friends and relatives in sub-Saharan Africa: A cross-sectional, multi-setting survey of knowledge, attitudes, and practices
Source: PLoS One. 2020 Mar 12;15(3):e0229565. doi: 10.1371/journal.pone.0229565 (PMC7067457; doi:10.1371/journal.pone.0229565)
Supplement: S1 Checklist — Checklist of reporting guidelines for observational studies. (PDF) [file pone.0229565.s001.pdf]

**Supplement 3: STROBE Statement Checklist for Cross-Sectional Studies**

Supplement to: Barriers to malaria prevention among immigrant travelers who visit friends and relatives in sub-Saharan Africa: A cross-sectional, multi-setting survey of knowledge, attitudes, and practices

| Section | Checklist item | Line number or description of location / inclusion in manuscript |
|---|---|---|
| **Title and abstract** | (a) Indicate the study's design with a commonly used term in the title or the abstract | Line 2: Cross-sectional survey |
| | (b) Provide in the abstract an informative and balanced summary of what was done and what was found | Lines 23-57: Includes section headers, rationale, approach, key findings, and significance. |
| **Introduction** | | |
| Background / rationale | Explain the scientific background and rationale for the investigation being reported | Lines 59-91: Background on VFRs and malaria in travelers, rationale for focus on VFR travelers to sub-Saharan Africa. |
| Objectives | State specific objectives, including any prespecified hypotheses | Lines 92-96: Specific objectives – identify differences in malaria prevention knowledge, attitudes, and practices among VFRs, and describe differences between VFRs and non-VFRs. |
| **Methods** | | |
| Study design | Present key elements of study design early in the paper | Lines 92-94: conducted a comprehensive community-based, cross-sectional survey of US travelers to malaria endemic countries in sub-Saharan Africa. |
| Setting | Describe the setting, locations, and relevant dates, including periods of recruitment, exposure, follow-up, and data collection | Malaria cases: lines 109-117<br>VFRs in the community: lines 118-138<br>Travel clinic patients: 139-147 |
| Participants | Give the eligibility criteria, and the sources and methods of selection of participants | Malaria cases: lines 109-117<br>VFRs in the community: lines 118-138<br>Travel clinic patients: 139-147 |
| Variables | Clearly define all outcomes, exposures, predictors, potential confounders, and effect modifiers. Give diagnostic criteria, if applicable | Lines 173-181: Describe variables<br>Supplemental Material 2: Provides complete assessment tools with all variables and their structure |
| Data sources / measurement | For each variable of interest, give sources of data and details of methods of assessment (measurement). Describe comparability of assessment methods if there is more than one group | Supplemental Material 2: Provides complete assessment tools with all variables and their structure<br>Lines 182-184 Comparability of assessment methods for groups |
| Bias | Describe any efforts to address potential sources of bias | Lines 213-214: interviewer training and rapport development<br>Lines 215-224: Reduction of bias through survey standardization across languages |
| Study size | Explain how the study size was arrived at | Lines 153-160 and Figure 2: Recruitment targets for each arm of the survey and Figure 2 |
| Quantitative variables | Explain how quantitative variables were handled in the analyses. If applicable, describe which groupings were chosen and why | Lines 229-237: dataset integration, rationale and explanation of groupings |
| Statistical methods | (a) Describe all statistical methods, including those used to control for confounding | Lines 244-253: Statistical methods, transformations for non-normality, significance level |
| | (b) Describe methods used to examine subgroups and interactions | Lines 244-253: Parametric and non-parametric comparative analyses |

| | | |
|---|---|---|
| | (c) Explain how missing data were addressed | Lines 262-385: All comparative analyses in results include sample n's to indicate missing data. |
| | (d) If applicable, describe analytical methods taking account of sampling strategy | Lines 234-237: Approach to combining responses across study arms, noting non-case-control sampling strategy (Figure 2) |
| | (e) Describe any sensitivity analyses | Not applicable |
| **Results** | | |
| Participants | (a) Report numbers of individuals at each stage of study—e.g. numbers potentially eligible, examined for eligibility, confirmed eligible, included in the study, completing follow-up, and analyzed | Line 161 – Figure 2<br>Line 262. |
| | (b) Give reasons for non-participation at each stage | Line 161 – Figure 2 eligibility and loss-to-follow up in the travel clinic arm |
| | (c) Consider use of a flow diagram | Line 161 – Figure 2 |
| Descriptive data | (a) Give characteristics of study participants (e.g .demographic, clinical, social), information on exposures / potential confounders | Line 279 – Table 1<br>Lines 262-269 |
| | (b) Indicate number of participants with missing data for each variable of interest | Lines 271-385: All comparative analyses in results include sample n's to indicate missing data. |
| Outcome data | Report numbers of outcome events or summary measures | Table 1, Table 2, Table 3 |
| Main results | (a) Give unadjusted estimates and, if applicable, confounder-adjusted estimates and their precision (e.g. 95% confidence interval). Make clear which confounders were adjusted for and why they were included | Lines 271-385<br>Table 1, Table 2, Table 3, Figure 4 |
| | (b) Report category boundaries when continuous variables were categorized | Line 279 - Table 1: trip count threshold at 5 trips, dichotomous education thresholds at grade school and high school cut points |
| | (c) If relevant, consider translating estimates of relative risk into absolute risk for a meaningful time period | Not applicable |
| Other analyses | Report other analyses done—e.g. analyses of subgroups and interactions, and sensitivity analyses | Lines 271-363: subgroup comparative analyses |
| **Discussion** | | |
| Key results | Summarize key results with reference to study objectives | Lines 393-405 |
| Limitations | Discuss limitations of the study, taking into account sources of potential bias or imprecision. Discuss both direction and magnitude of any potential bias | Lines 480-512 |
| Interpretation | Give a cautious overall interpretation of results considering objectives, limitations, multiplicity of analyses, results from similar studies, and other relevant evidence | Lines 407-429, lines 514-533 |
| Generalizability | Discuss the generalizability (external validity) of the study results | Lines 489-493 |
| **Other information** | | |
| Funding | Give the source of funding and the role of the funders for the present study and, if applicable, for the original study on which the present article is based | Lines 254-259. |
