# Supplementary material for: Barriers to malaria prevention among immigrant travelers in the United States who visit friends and relatives in sub-Saharan Africa: A cross-sectional, multi-setting survey of knowledge, attitudes, and practices
Source: PLoS One. 2020 Mar 12;15(3):e0229565. doi: 10.1371/journal.pone.0229565 (PMC7067457; doi:10.1371/journal.pone.0229565)
Supplement: S1 Document — Surveys and consent materials are included for the five survey versions used across the three study arms. Materials are ordered as follows: S2.1) Consent and Survey– 2016 Case Interview—Pilot Instrument; S2.2) Consent and Survey–Final Case Interview; S2.3) Consent and Survey–Emergency Department; S2.4) Consent and Survey–Community Paper and Online; S2.5) Consent and Survey–Travel Clinic. (PDF) [file pone.0229565.s003.pdf]

S2 Document. Survey Material Packet.

Survey questionnaires and consent materials are included for the five survey versions used across the three study arms. Materials are ordered as follows:

S2.1) Consent and Survey – 2016 Case Interview - Pilot Instrument: Pages 2-16

S2.2) Consent and Survey – Final Case Interview: Pages 17-30

S2.3) Consent and Survey – Emergency Department: Pages 31-39

S2.4) Consent and Survey – Community Paper and Online: Pages 40-48

S2.5) Consent and Survey – Travel Clinic: Pages 49-60

MALARIA PATIENT INTERVIEW FORM: *(as of 11/7/2016)*

Introductory Narrative and Tennessen Warning

The following text does not need to be followed verbatim, but you do need to touch on all of the following points:

INTRODUCTION

Ask for case-patient (if ≥ 18 years old) or parent/guardian (if < 18 years)

- *Under 12 years old: Conduct interview with parent/guardian.*
- *12-17 years old: You can conduct interview with the patient, if parent/guardian allows, but make sure to obtain parent/guardian's consent first. The child will also need to assent to the interview.*
- *Adults ≥ 18 years old: It is preferable to interview the case-patient. Alternatively, it is okay to interview the spouse or another very close family member, IF the case-patient will be difficult to get a hold of and if the family member indicates a good understanding of their history.*

Introduction

- I'm calling from the mosquito-borne disease unit at the Minnesota Department of Health to follow up on your recent malaria infection. It looks like you tested positive for malaria back in [month or season].
- We are trying to interview cases of malaria to learn about what people already know about malaria and learn more about how the disease affected you. The questions will ask about what you know about malaria and what you've learned about malaria since becoming sick. This information will help us to better prepare people who visit areas where malaria is found.
- This interview will take about 15-20 minutes. Does that sound okay/would now be a good time?
    - *If yes, read Tennessen.*
    - *If no, find out a better time to call back. Mark down as LTF if they clearly refuse.*

TENNESSEN WARNING

- **Why** we are calling: covered above
- All identifying information is **private** at MDH and is only seen by MDH staff that work with malaria. We do not share any identifying information with anyone else. Summary information, like where people traveled when they got malaria, or how many cases occurred in Minnesota residents, are shared but none of this information could be used to identify any individuals.
- This interview is **voluntary**. You can refuse to answer any question or stop me at any time.

LEAVING A MESSAGE

Voice mail

- Leave a message after the 2nd attempt. For more a detailed explanation on when to leave a message, please refer to the 'Exposure Call Protocol.' *Do not explicitly state that you are calling about John Doe's malaria.*
    - This is a message for John Doe. This is [your name] calling from the Minnesota Department of Health. I'm with the mosquito-borne disease unit here and am hoping we can chat sometime soon. Please give me a call at 651-201-xxxx when you have a chance. We look forward to speaking with you. Thanks!

Leaving message with a person (case-patient not available)

- I'm calling from the mosquito-borne disease unit at the MN Department of Health. Are you a family member of John's?
    - *If spouse or very close adult family member:* Do similar intro as if you are speaking with the case-patient and find out when would be a good time to call back, or leave your phone number. Alternatively, you can try to interview the family member if the person feels they know a lot about the case-patient's malaria experience.
    - *If person who answers is a child or isn't a close family member:* I'm calling from the mosquito-borne disease unit at the Minnesota Department of Health. Do you know when would be a good time to get a hold of John? … I will try to call back then. May I also leave a message and give you our phone number here?

**Patient Information:**	**Name** *(last, first)*: ____
	**DOB:** _____________
**Interview Outcome:**	**Date:** ____/____/__________ **Interviewer:** ________________
	☐ **Tennessen**
	☐ **Patient** ☐ **Proxy (name and relationship):** ______________________________

Refer to 'Malaria Interview Introductory Narrative' for introductory script.

**** Note to interviewer: unless instructed to do so below, do <u>not</u> read answer options to respondents.**

Section 1: Malaria Knowledge

I want to understand what you already know about malaria. I'll read you a few statements and ask you to rate them on the following scale:

1 – Not true 2 – Rarely true 3 – Sometimes true 4 – Mostly/usually true 5 – Very true/always true

1. **<u>Thinking about before you got malaria, how true are the following statements?</u>**

   a) I was concerned for myself about getting malaria
   1 2 3 4 5

   b) When traveling to areas with malaria, my children are more likely to become sick from malaria than I am
   1 2 3 4 5

   c) Malaria is only serious in children
   1 2 3 4 5

   d) Malaria could be a deadly disease for me
   1 2 3 4 5

   e) It is important to take precautions when I travel to countries where there is malaria
   1 2 3 4 5

2. How do people get malaria? (*check all that are mentioned and write in others*)

   ☐ From mosquitoes
   ☐ From water
   ☐ Other (please describe):
   ☐ From bad air
   ☐ From another person

3. Can people catch malaria directly from another person?

   ☐ Yes
   ☐ No

4. Can you prevent malaria?

☐ Yes

If yes, how can you prevent malaria? *(check all that apply)*

☐ Immunizations/shots
☐ Bed nets
☐ Insect repellent
☐ Take medications
☐ Other (describe):

☐ No

Section 2: Previous Malaria Exposure and Infection

5. Is this the first time you had malaria?

☐ Yes
☐ No
☐ Don't know/undiagnosed

a. How many times before? ____

b. When was the last time you had malaria? _______________________________________

c. Last time, how did you know it was malaria? (i.e., who diagnosed you?)

☐ Medical Provider
☐ I figured it out myself
☐ Other: ______________________________

d. Is that how you usually do it?

☐ Yes
☐ No
☐ Other: ____

e. Were you treated after your diagnosis?

☐ Yes – if **yes**, do you remember what medications/treatments you were given?
____
☐ No

Section 3: Current Trip

Now I'd like to find out more about your most recent trip … *[prompt with travel dates from CRF]*

6. What was the main reason for your travel?

☐ Visit friends or relatives
☐ Business
☐ Vacation – not visiting friends or relatives
☐ Studying abroad / research
☐ Mission trip
☐ Other: ____

7. Where did you travel to? *(List specific country[ies], city[ies])*

8. How long were you there? (days, weeks, months)

9. Did you or anyone traveling with you talk to someone about malaria before you left?

   - ☐ Yes **– if yes, go to 9a.**
   - ☐ No **– skip to question 10**

   9a. **If <u>yes</u>**, who did you/they talk to?

   - ☐ Family member
   - ☐ Community elder
   - ☐ Healthcare provider
   - ☐ Religious leader
   - ☐ Travel agent
   - ☐ Other: ____________________________

   If it was a **<u>healthcare provider</u>**, was it your regular healthcare provider, travel clinic, or somewhere else?

   - ☐ Your regular healthcare provider (primary care)
   - ☐ At a travel clinic
   - ☐ Other: ____

   9b. If you traveled as a family, did all the family members see a health care provider before travel or only specific family members? Which ones?

   - ☐ N/A (traveled alone)
   - ☐ All travelers
   - ☐ Only children
   - ☐ A pregnant traveler
   - ☐ Other: ____

10. During your most recent trip to _________ when you got malaria, did you use insect repellent?

   - ☐ Yes
   - ☐ No
   - ☐ Sometimes

   10a. If **<u>yes/sometimes</u>**, when did you use it?

   - ☐ Applied in morning when getting ready
   - ☐ Only when I saw mosquitoes
   - ☐ When I knew I would be outside
   - ☐ Throughout the day
   - ☐ Other: ___________________
   - ☐ Before I went to bed
   - ☐ Only if I saw mosquito bites on my skin
   - ☐ When I was outside after sundown
   - ☐ If other people complained of mosquitoes

   10b. If **<u>yes/sometimes</u>**, do you remember what kind of repellent?

   10c. **Were there any other times you used repellent?** *(ask again to prompt and use check boxes above)*

11. Did you use anything else to keep mosquitoes away?

- ☐ Yes (if yes, please describe):

- ☐ No

12. During your most recent trip to _________ when you got malaria, did you sleep underneath a bed net?
- ☐ Yes **– go to question 12a.**
- ☐ No **– go to question 12b.**

12a. Did you use the bed net all of the time or only in some situations?

- ☐ All the time
- ☐ Other (please describe):

12b. If you did not use a bed net, why not? *(check all that apply)*:

- ☐ I did not think I needed a bed net/to sleep under a bed net
- ☐ I did not know where to buy a net
- ☐ I do not like sleeping under a net
- ☐ I gave my net to someone else to use/not enough nets for everyone
- ☐ I slept in a place in which a bed net was unnecessary

13. When you last traveled, were any special health precautions recommended to you?
(*Read list to case and select all that apply)*

- ☐ Vaccines
- ☐ Medications for malaria
- ☐ Medications for diarrhea
- ☐ Other (please describe):
- ☐ Hand sanitizer
- ☐ Insect repellents
- ☐ Bed nets

13a. Who recommended these to you?

Section 4: Current illness

Now I'd like to ask you some more questions about how malaria affected you. For your most recent/current illness:

14. When did you start feeling sick? *(test/doctor's visit was on [lab date])* Estimated date: ___________

15. When you started to feel sick were you abroad, traveling between countries/cities, or back in the US?

- ☐ In another country
- ☐ Traveling between countries/cities
- ☐ Back in the U.S.

16. Before you traveled this time, were you prescribed a malaria prevention medication?

- ☐ Yes
- ☐ No – **skip to question 18**

16a. **<u>If yes</u>**, who prescribed the medication?

- ☐ Regular doctor
- ☐ Travel clinic
- ☐ Pharmacist (didn't see a provider)
- ☐ Other: ______________________________

16b. **<u>If yes</u>**, did you pick up the prescription?

- ☐ Yes
- ☐ No – **go to question 18**

16c. **<u>If yes</u>**, did you take the malaria prevention medication?

- ☐ Yes
- ☐ No

17a. Do you remember what medication(s) you received?

- ☐ Chloroquine (Aralen®)
- ☐ Atovaquone-proguanil (Malarone®)
- ☐ Don't remember
- ☐ Mefloquine (Lariam ®)
- ☐ Doxycycline
- ☐ Other: ______________________________

<u>If don't remember the name of the medicine, do you remember…</u>

Was it once a day or once a week?

- ☐ Once a day
- ☐ Once a week

The shape of the pill?

- ☐ Liquid
- ☐ Oval
- ☐ Circle

Color of pill?

If you break it open, was there powder inside?

- ☐ Powder inside (capsule)
- ☐ Pill was same texture inside and out (tablet)

17b. How often were you supposed to take it?

- ☐ Once a day
- ☐ Once a week
- ☐ Don't know

17c. After you got back, how long did you take it?

- ☐ Stopped right when I got back
- ☐ For <3 days after I got back
- ☐ 1 week after I got back
- ☐ 2 weeks after I got back
- ☐ 3 weeks after I got back
- ☐ 4 weeks after I got back

17d. Do you remember how long were you supposed to take it after you got back?

- ☐ Stop right when I got back
- ☐ For <3 days after I got back
- ☐ 1 week after I got back
- ☐ 2 weeks after I got back
- ☐ 4 weeks after I got back
- ☐ Can't remember

17e. Did you take it as prescribed or did you miss or skip any doses?

- ☐ As prescribed
- ☐ Missed/skipped doses
  - ☐ Missed one
  - ☐ Missed a few days
  - ☐ Missed a lot of days
- ☐ I stopped taking it

17f. If you skipped or missed doses or stopped taking it, what were the reasons?

- ☐ Forgot to take medicine
- ☐ The pharmacy didn't give me enough
- ☐ Lost it
- ☐ Had side effects (describe): ____
- ☐ Other: ____

18a. If medications were not prescribed **OR** if medications were prescribed but you didn't take them, why not?

[*Not prescribed only*:]

- ☐ No one told me I needed preventive medications
- ☐ Didn't know there were preventive medications

[*Prescribed, didn't take it only*:]

- ☐ Could not get medication at pharmacy/pharmacy did not stock medication

[*Either*:]

- ☐ It was too expensive
- ☐ Not a serious disease
- ☐ I would rather take my chances
- ☐ Don't think I needed it
- ☐ The medication has too many side effects/am worried about side effects
- ☐ I planned to just buy medicine if I got sick while traveling/easier to just treat if you get sick
- ☐ My trip was too long for me to take a prevention medication
- ☐ They didn't give me enough for my whole trip
- ☐ My insurance didn't cover it
- ☐ I forgot

- ☐ I didn't think there was malaria where I was going
- ☐ I planned to get it when I got to my destination
- ☐ It wasn't the rainy season
- ☐ Medications do not work well
- ☐ I don't like to take medications
- ☐ Other: ____

18b. Are there any other reasons that you didn't take medication? (ask question again and use options above)

Section 5: Traveling with children

19. On your last trip did any children travel with you?

- ☐ Yes – if yes, how many? __________________________
- ☐ No – **skip to question 23**

20. Before you traveled, was the child prescribed a malaria prevention medication?

- ☐ Yes
- ☐ No – **skip to question 22**

20a. **If yes**, did someone pick up the prescription?

- ☐ Yes
- ☐ No – **skip to question 22**

20b. **If yes**, did the child take the malaria prevention medication?

- ☐ Yes
- ☐ No – **skip to question 22**

21. a. Do you know what medication/s the child received?

- ☐ Chloroquine (Aralen®)
- ☐ Atovaquone-proguanil (Malarone®)
- ☐ Don't remember
- ☐ Mefloquine (Lariam ®)
- ☐ Doxycycline
- ☐ Other: ________________________

If don't remember the name of the medicine, do you remember…

a. Was it once a day or once a week?

- ☐ Once a day
- ☐ Once a week

b. Shape of the pill?

- ☐ Liquid
- ☐ Oval
- ☐ Circle

c. Color of pill?

d. If you break it open, was there powder inside?

- ☐ Powder inside (capsule)
- ☐ Pill was same texture inside and out (tablet)

21b. How often was the child supposed to take it?

- ☐ Once a day
- ☐ Once a week
- ☐ Don't know

21c. After you got back, how long did the child take it?

- ☐ Stopped right when child got back
- ☐ For <3 days after child got back
- ☐ 1 week after child got back
- ☐ 2 weeks after child got back
- ☐ 4 weeks after child got back

21d. How long was the child supposed to take it after he/she got back?

- ☐ Stop right when child got back
- ☐ For <3 days after child got back
- ☐ 1 week after child got back
- ☐ 2 weeks after child got back
- ☐ 4 weeks after child got back
- ☐ Can't remember

21e. Did the child take it as prescribed on the label or did he/she miss or skip any doses?

- ☐ As prescribed
- ☐ Missed/skipped doses
  - ☐ Missed one
  - ☐ Missed a few days
  - ☐ Missed a lot of days
- ☐ I stopped taking it

21f. If he/she skipped or missed doses or stopped taking it, what were the reasons?

- ☐ Forgot to take medicine
- ☐ The pharmacy didn't give me enough
- ☐ Lost it
- ☐ Had side effects (describe): ____
- ☐ Other: ____

22a. If medications were not prescribed **OR** if medications were prescribed but he/she didn't take the medication, why not?

[*Not prescribed only*:]

- ☐ No one told me preventive medications were needed
- ☐ Didn't know there were preventive medications

[*Prescribed, didn't take it only*:]

- ☐ Could not get medication at pharmacy / pharmacy did not stock medication

[*Either*:]

- ☐ It was too expensive
- ☐ Not a serious disease
- ☐ I would rather take my chances
- ☐ Don't think child needed it
- ☐ The medication has too many side effects/worried about side effects in children
- ☐ I planned to just buy medicine if child got sick while traveling/easier to just treat if you get sick
- ☐ My trip was too long for child to take a prevention medication
- ☐ They didn't give child enough for whole trip
- ☐ My insurance didn't cover it
- ☐ I forgot to make sure child took
- ☐ I didn't think there was malaria where we were going
- ☐ I planned to get it when we got to our destination
- ☐ It wasn't the rainy season
- ☐ Medications do not work well
- ☐ I don't like to take medications/give medications to children
- ☐ Other: ____

22b. Are there any other reasons the child didn't take medication? (ask question again and use options above)

Section 6: Post-travel questions

My next set of questions is about when you got back from your recent trip to _________:

23. When you started to feel sick/first noticed your symptoms, what did you think was making you sick?

- ☐ Malaria
- ☐ Something else (describe): ____

24. When you noticed your symptoms, where was the first place you looked for information/advice about your illness?

- ☐ Family or friend abroad
- ☐ Healthcare provider abroad
- ☐ Family or friend in US
- ☐ Primary care in US
- ☐ Urgent care/ER in US
- ☐ Travel clinic in US
- ☐ Internet
- ☐ Other: ___________________________

25. Did you try to treat yourself (or your child) before seeking medical care?

- ☐ Yes
- ☐ No

25a. If **yes**, why? (*Check all that apply*):

- ☐ Doctors in the US don't know about malaria
- ☐ Going to the doctor is too expensive
- ☐ I knew it was malaria and how to treat it
- ☐ I already had the medicine
- ☐ Buying medicine is too expensive
- ☐ Didn't know it was malaria
- ☐ Malaria is easy to treat
- ☐ Malaria is not a serious problem
- ☐ Other: ____

25b. If **yes**, what did you do to treat yourself? (*Check all that apply*):

- ☐ Took medication for fever and/or pain? (e.g. paracetamol, Tylenol, ibuprofen)
  If **yes,** what medicine: ____
- ☐ Herbal remedy
- ☐ Other: ____

25c. If **yes**, did you take a medicine specifically for malaria?

- ☐ Yes
  If yes, what medicine? ____
  Where was the medicine from/where did you get it? _________________________
- ☐ No

26. After returning from any trip to a country with malaria, have you ever self-treated a child in your family for malaria before going to a doctor?

- ☐ Yes – **skip to question 25a.**
- ☐ No – **skip to question 25b.**

26a. If yes, why? (*Check all that apply*):

- ☐ Malaria is easy to treat
- ☐ Malaria is not a serious problem
- ☐ Didn't know it was malaria
- ☐ I already had the medicine
- ☐ Buying medicine is too expensive
- ☐ Going to the doctor is too expensive
- ☐ I knew it was malaria and how to treat it
- ☐ Doctors in the US don't know about malaria
- ☐ Other:

26b. If no, why not? *(please explain)*:

27. How many health professionals did you see or visits to clinics, urgent care, emergency departments did you have before you were told that malaria was your diagnosis?

- ☐ One
- ☐ Two
- ☐ Three
- ☐ Four or more
- ☐ Never told I had malaria

Section 7: Future plans

28. Would you seek medical advice before traveling outside the U.S. if you might be going to a malaria area?

- ☐ Yes -- If **yes**; where would you get this advice (Check all that apply)?
  - ☐ Internet
  - ☐ Travel agent
  - ☐ Pharmacy
  - ☐ Urgent care
  - ☐ Primary care
  - ☐ Travel clinic
  - ☐ Other: ______________________________
- ☐ No -- If **no**, why not? (Check all that apply)
  - ☐ I don't have insurance
  - ☐ Rather take my chances
  - ☐ Too busy
  - ☐ Not worried about malaria/don't think I can get malaria ("I am immune")
  - ☐ Other:

29. The next time you (or your child) travel to a country with malaria, would you take malaria prevention medication if it was suggested to you?

- ☐ Yes
- ☐ No

If **no**, why not?

- ☐ Too expensive
- ☐ Not a serious disease
- ☐ I would rather take my chances
- ☐ Don't think I'll need it
- ☐ I will probably forget
- ☐ I don't like to take medications
- ☐ My insurance doesn't cover it
- ☐ Medications do not work well
- ☐ It won't be the rainy season
- ☐ Can't get enough medication for my whole trip
- ☐ I don't think there is malaria where I'll travel
- ☐ I will get it when I got to my destination
- ☐ The medication has too many side effects/am worried about side effects
- ☐ I plan to just buy medicine if I get sick while traveling/easier to treat if you get sick
- ☐ My trip will be too long for me to take a prevention medication
- ☐ I can't find a pharmacy that stocks the medication
- ☐ Other:

30. The next time you travel to a country with malaria, would you use a bed net if it were recommended to you?

- ☐ Yes
- ☐ No – if no, why not?

31. The next time you travel to a country with malaria, would you use mosquito repellent if it were recommended to you?

    - ☐ Yes
    - ☐ No – if no, why not?

32. The next time you travel to a country with malaria, would you use insecticide-treated clothing if it were recommended to you?

    - ☐ Yes
    - ☐ No – if no, why not?

33. Is there anything you know about malaria now that you wish you had known before you got sick?

34. In your opinion, what would be the best way to educate people about malaria?

Additional Demographic Information

35. Gender:

- ☐ Male
- ☐ Female

36. Were you born in the United States?

- ☐ Yes
- ☐ No

35a. **If no**, where were you born? (Record country of birth): ______________________________

37. Have you ever lived in a country other than the United States or the country you were born?

- ☐ Yes – where? ____
- ☐ No

38. Have you ever lived in an area that has malaria? (Not recent trip, but lived there)

- ☐ Yes
- ☐ No – **skip to question 39**

38a. When was the last time?

38b. Where did you live?

38c. How long did you live there? ________________ (weeks, months, years)

38d. How long have you lived in the US? ___________________ (weeks, months, years)

39. Where were your parents born?

Mom: _______________________________

Dad: ________________________________

40. How would you describe your race/ethnicity?:

____

41. Before this most recent trip, how long ago did you last travel to a country with malaria?

42. What is the highest level of educational you completed?

- ☐ Grade school
- ☐ High School
- ☐ Associate Degree
- ☐ Bachelor's Degree
- ☐ Graduate School

36. Additional notes____

S2.2) Consent and Survey – Final Case Interview

MALARIA PATIENT INTERVIEW FORM: *(as of 11/7/2016)*

Introductory Narrative and Tennessen Warning

The following text does not need to be followed verbatim, but you do need to touch on all of the following points:

INTRODUCTION
**Ask for case-patient (if ≥ 18 years old) or parent/guardian (if < 18 years)**<br>• *Under 12 years old: Conduct interview with parent/guardian.*<br>• *12-17 years old: You can conduct interview with the patient, if parent/guardian allows, but make sure to obtain parent/guardian's consent first. The child will also need to assent to the interview.*<br>• *Adults ≥ 18 years old: It is preferable to interview the case-patient. Alternatively, it is okay to interview the spouse or another very close family member, IF the case-patient will be difficult to get a hold of and if the family member indicates a good understanding of their history.*<br><br>**Introduction**<br>• I'm calling from the mosquito-borne disease unit at the Minnesota Department of Health to follow up on your recent malaria infection. It looks like you tested positive for malaria back in [month or season].<br>• We are trying to interview cases of malaria to learn about what people already know about malaria and learn more about how the disease affected you. The questions will ask about what you know about malaria and what you've learned about malaria since becoming sick. This information will help us to better prepare people who visit areas where malaria is found.<br>• This interview will take about 15-20 minutes. Does that sound okay/would now be a good time?<br>o *If yes, read Tennessen.*<br>o *If no, find out a better time to call back. Mark down as LTF if they clearly refuse.*

TENNESSEN WARNING
• **Why** we are calling: covered above<br>• All identifying information is **private** at MDH and is only seen by MDH staff that work with malaria. We do not share any identifying information with anyone else. Summary information, like where people traveled when they got malaria, or how many cases occurred in Minnesota residents, are shared but none of this information could be used to identify any individuals.<br>• This interview is **voluntary**. You can refuse to answer any question or stop me at any time.

LEAVING A MESSAGE
**Voice mail**<br><br>• Leave a message after the 2nd attempt. For more a detailed explanation on when to leave a message, please refer to the 'Exposure Call Protocol.' *Do not* *<u>explicitly</u>* *state that you are calling about John Doe's malaria.*<br><br>○ This is a message for John Doe. This is [your name] calling from the Minnesota Department of Health. I'm with the mosquito-borne disease unit here and am hoping we can chat sometime soon. Please give me a call at 651-201-xxxx when you have a chance. We look forward to speaking with you. Thanks!<br><br>**Leaving message with a person (case-patient not available)**<br><br>• I'm calling from the mosquito-borne disease unit at the MN Department of Health. Are you a family member of John's?<br><br>○ *<u>If spouse or very close adult family member:</u>* Do similar intro as if you are speaking with the case-patient and find out when would be a good time to call back, or leave your phone number. Alternatively, you can try to interview the family member if the person feels they know a lot about the case-patient's malaria experience.<br><br>○ *<u>If person who answers is a child or isn't a close family member:</u>* I'm calling from the mosquito-borne disease unit at the Minnesota Department of Health. Do you know when would be a good time to get a hold of John? … I will try to call back then. May I also leave a message and give you our phone number here?

**Patient Information:**	**Name** *(last, first)*: ____
	**DOB:** ____________
**Interview Outcome:**	**Date:** ____/____/_________ **Interviewer:** _______________
	☐ **Tennessen**
	☐ **Patient** ☐ **Proxy (name and relationship):** ____________________________

Refer to 'Malaria Interview Introductory Narrative' for introductory script.

***** Note to interviewer: unless instructed to do so below, do <u>not</u> read answers to respondents. *****

Section 1: Malaria Knowledge

I want to understand what you know about malaria. I'll read you a few statements and ask you to rate them on a scale of 1 to 5:

1 – Not true **3 – Somewhat true** **5 – Very true/always true**

1. Thinking about **<u>before you got malaria</u>**, how true are the following statements?

   f) I was concerned for myself about getting malaria when traveling

   1 2 3 4 5

   g) Malaria can be a deadly disease

   1 2 3 4 5

2. When traveling to areas with malaria, who is more likely to become sick from malaria – you or your child?
   ☐ You / Adult
   ☐ Child
   ☐ Equally likely

3. How do people get malaria? (*check all that are mentioned and write in others*)
   ☐ From mosquitoes
   ☐ From bad air
   ☐ From drinking water
   ☐ From another person ***<u>(prompt with clarifier #4)</u>***
   ☐ Other (please describe): ____

4. Can people catch malaria directly from another person who is sick with malaria?
   Directly means spreading from one person right to another.

   ☐ Yes
   ☐ No

5. Can you prevent malaria?

   ☐ Yes

If <u>yes</u>, how can you prevent malaria? *(check all that the participant lists)*
   ☐ Immunizations/shots
   ☐ Bed nets
   ☐ Insect repellent
   ☐ Take medications
   ☐ Other (describe): ____

   ☐ No

Section 2: Previous Malaria Exposure and Infection

6. Is this the first time you had malaria?
   - ☐ Yes
   - ☐ No
   - ☐ Don't know

   b. How many times before? ____

   c. When was the last time you had malaria? _______________________________________

   d. Last time, how did you know it was malaria? (i.e., who diagnosed you?)
   - ☐ Medical Provider
   - ☐ I figured it out myself / recognized symptoms of malaria
   - ☐ Other: ________________________________

   e. ***If person has had malaria <u>more than once</u> before:*** Is that how you usually knew you had malaria?
   - ☐ Yes
   - ☐ No – How would you know it was malaria? ______________________________________

   f. Were you treated with medicine last time you had malaria?

   - ☐ Yes – if **<u>yes</u>**, do you remember what medications/treatments you were given?

____

   - ☐ No

Section 3: Current Trip

Now I'd like to find out more about your most recent trip … *[prompt with travel dates from CRF]*

7. What were the main reasons for your travel?

If more than one, rank primary (#1), secondary reasons (#2), etc.

_______ Visit friends or relatives
_______ Business
_______ Vacation – not visiting friends or relatives
_______ Studying abroad / research
_______ Mission trip
_______ Other: ____

8. Where did you travel to? *(List specific country[ies])* ____

9. How long were you on your trip? ____

10. Did you talk to someone about malaria before you left?
    - ☐ Yes **– if yes, go to 10a**
    - ☐ No **– skip to question 11**

10a. **If <u>yes</u>**, who did you/they talk to?

- ☐ Family member / friend
- ☐ Community elder
- ☐ Healthcare provider
  (check 11 as Yes, skip to 11a)
- ☐ Religious leader
- ☐ Travel agent
- ☐ Other: ____________________________

11. ***If participant does not list healthcare provider above:*** Did you see a healthcare provider before you left?
    - ☐ **Yes – Go to 11a**
    - ☐ **No – Why not?** ____

11a. **<u>If yes:</u>** Was it your regular healthcare provider, travel clinic, or somewhere else?

- ☐ Regular healthcare provider (primary care)
- ☐ Travel clinic
- ☐ Both regular healthcare provider and travel clinic
- ☐ Urgent care
- ☐ Pharmacy / minute clinic
- ☐ Other: ______________________________________

12. Did you travel with family? ***If yes, continue question, if no go to question 13***
    - ☐ Yes
    - ☐ No, did not travel with family

12a. **<u>If yes</u>** Did everyone in the family who traveled see a healthcare provider?

- ☐ Yes
- ☐ No
- ☐ Some were seen → Who? ______________________________________

12b. Were you more concerned about making sure certain family members were seen by a healthcare provider than others?

- ☐ No
- ☐ Yes → Who? ______________________________________

12c. Why?

- ☐ Only certain family members have insurance
- ☐ Cost
- ☐ Risk of Illness
- ☐ Age (very young or old)
- ☐ Pregnancy
- ☐ Other: ______________________________________

13. During your most recent trip did you use any of the following strategies to keep mosquitos away or stay healthy? ***I'm going to list a few examples and you can respond: yes, no, or sometimes***

Did you sleep under bed nets? ☐ **Yes** ☐ **No** ☐ **Sometimes**

<u>If sometimes</u>, when? ______________________________________

<u>If no,</u> why not?

- ☐ I did not think I needed a bed net/to sleep under a bed net
- ☐ I did not know where to buy a net
- ☐ I do not like sleeping under a net
- ☐ I gave my net to someone else to use/not enough nets for everyone
- ☐ I slept in a place in which a bed net was unnecessary
- ☐ Other: ______________________________________

Did you use repellent / creams /sprays / lotions? ☐**Yes** ☐**No** ☐**Sometimes**

If yes / sometimes, when did you wear repellent?

- ☐ Applied in morning when getting ready
- ☐ Only when I saw mosquitoes
- ☐ When I knew I would be outside
- ☐ Throughout the day
- ☐ Before I went to bed
- ☐ Only if I saw mosquito bites on my skin
- ☐ When I was outside after sundown
- ☐ If other people complained of mosquitoes
- ☐ Other: ____

If yes / sometimes, do you know what the active ingredient was?

- ☐ Deet
- ☐ Picaridin
- ☐ Other: ____

☐ Yes	☐No	☐Sometimes	Mosquito coils
☐ Yes	☐No	☐Sometimes	Environmental cleanup e.g removing sources of standing water
☐ Yes	☐No	☐Sometimes	Staying indoors when mosquitos are out
☐ Yes	☐No	☐Sometimes	Wear long clothing
☐ Yes	☐No	☐Sometimes	Practice good food safety / hygiene
☐ Yes	☐No	☐Sometimes	Ensure clean water source (safe, bottled)
☐ Yes	☐No	☐Sometimes	Educate yourself on risks of malaria before traveling
☐ Yes	☐No	☐Sometimes	Pick where you stayed in order to avoid mosquitos (AC/screens)
☐ Yes	☐No	☐Sometimes	Get vaccinations

Anything else? ____

Section 4: Current illness

Now I'd like to ask you some more questions about how malaria affected you. For your most recent/current illness:

14. When did you start feeling sick? *(test/doctor's visit was on [lab date])* Estimated date: ___________

15. When you started to feel sick were you abroad, traveling between countries/cities, or back in the US?
    - ☐ In another country
    - ☐ Traveling between countries/cities
    - ☐ Back in the U.S.

16. Did you take a malaria prevention medicine during your last trip?
    - ☐ Yes
    - ☐ No
    - ☐ Sometimes

17. Did a healthcare provider write you a prescription for a malaria prevention medicine?
    - ☐ Yes
    - ☐ No – **If 16 & 17 are No, skip to 23**

17a. **<u>If yes,</u>** did you pick up the medicine in the US or once you arrived to your destination?

- ☐ Picked up the medicine in the US

<u>If in the US:</u> Who prescribed the medication?

- ☐ Regular doctor
- ☐ Pharmacist (didn't see a provider)
- ☐ Travel clinic
- ☐ Other: ______________________________
- ☐ Picked up the prescription in destination country

17b. **If 16 is yes/sometimes and 17 is no, clarify:** Did you buy the malaria prevention medicine without a prescription once you arrived to your destination?

- ☐ Yes
- ☐ No – Explain: ____

18. Do you remember what medication(s) you received?
    - ☐ Chloroquine (Aralen®)
    - ☐ Mefloquine (Lariam ®)
    - ☐ Atovaquone-proguanil (Malarone®)
    - ☐ Doxycycline
    - ☐ Don't remember
    - ☐ Other: ________________________________

18a-d. **<u>If don't remember the name of the medicine, do you remember…</u>**

a. Was it once a day or once a week?

- ☐ Once a day
- ☐ Once a week

b. The shape of the pill?

- ☐ Liquid
- ☐ Oval
- ☐ Circle

c. Color of pill?

d. If you break it open, was there powder inside?

- ☐ Powder inside (capsule)
- ☐ Pill was same texture inside and out (tablet)

19. How often were you supposed to take it?
    - ☐ Once a day
    - ☐ Once a week
    - ☐ Don't know

20. Did you take it as prescribed or did you miss or skip any doses?
    - ☐ No, I took it as prescribed
    - ☐ Missed/skipped doses
        - ☐ Missed one
        - ☐ Missed a few days
        - ☐ Missed a lot of days
    - ☐ I stopped taking it

20a. **<u>If you skipped or missed doses or stopped taking it</u>**: What were the reasons?

- ☐ Forgot to take medicine
- ☐ The pharmacy didn't give me enough
- ☐ Lost it
- ☐ Had side effects (describe): ____
- ☐ Other: ____

21. Do you remember how long were you supposed to take it after you got back?
    - ☐ Stop right when I got back
    - ☐ For <3 days after I got back
    - ☐ 1 week after I got back
    - ☐ 2 weeks after I got back
    - ☐ 4 weeks after I got back
    - ☐ Can't remember

22. After you got back, how long did you take it?
    - ☐ Stopped right when I got back
    - ☐ For <3 days after I got back
    - ☐ 1 week after I got back
    - ☐ 2 weeks after I got back
    - ☐ 3 weeks after I got back
    - ☐ 4 weeks after I got back

23. **<u>If you didn't take medications,</u>** why not?

[*Not prescribed only*:]
- ☐ No one told me I needed preventive medications
- ☐ Didn't know there were preventive medications

[*Prescribed, didn't take it only*:]
- ☐ Could not get medication at pharmacy/pharmacy did not stock medication

[*Either*:]
- ☐ It was too expensive
- ☐ Not a serious disease
- ☐ I would rather take my chances
- ☐ Don't think I needed it
- ☐ The medication has too many side effects/am worried about side effects
- ☐ I planned to just buy medicine if I got sick while traveling/easier to just treat if you get sick
- ☐ My trip was too long for me to take a prevention medication
- ☐ They didn't give me enough for my whole trip
- ☐ My insurance didn't cover it
- ☐ I forgot
- ☐ I didn't think there was malaria where I was going
- ☐ I planned to get it when I got to my destination
- ☐ It wasn't the rainy season
- ☐ Medications do not work well
- ☐ I don't like to take medications
- ☐ Other: ____

23b. Are there any other reasons that you didn't take medication? (ask question again and use options above)

Section 5: Traveling with Children

24. On your last trip did any children travel with you?
    - ☐ Yes – if yes, how many? ________________________ How old were they? ______________
    - ☐ No – **skip to question 33**

25. Did your child/ren take a malaria prevention medicine during your last trip?
    - ☐ Yes →Did the child finish taking the medicine after he/she got back? ☐ Yes ☐ No ☐ Don't recall
    - ☐ No
    - ☐ Sometimes→Did the child finish taking the medicine after he/she got back? ☐ Yes ☐ No ☐ Don't recall

26. Did a healthcare provider write your child/ren a prescription for a malaria prevention medicine?
    - ☐ Yes
    - ☐ No – **If 25 & 26 are No, skip to 32**

26a. **<u>If yes,</u>** did you pick up the medicine for your child/ren in the US or once you arrived to your destination?

☐ Got the medicine in the US

<u>If in the US:</u> Who prescribed the medication?

☐ Regular doctor ☐ Pharmacist (didn't see a provider)
☐ Travel clinic ☐ Other: _______________________________

☐ Got the prescription in destination country

26b. **If 25 is yes/sometimes and 26 is no, clarify:** So you bought the malaria prevention medicine for your child/ren without a prescription once you arrived to your destination?

☐ Yes.
☐ No – Explain: ____

27. Do you remember what medication(s) you received for your child/ren?
    - ☐ Chloroquine (Aralen®)
    - ☐ Atovaquone-proguanil (Malarone®)
    - ☐ Don't remember
    - ☐ Mefloquine (Lariam ®)
    - ☐ Doxycycline
    - ☐ Other: _________________________________

27a-d. **<u>If don't remember the name of the medicine, do you remember…</u>**

a. Was it once a day or once a week?
- ☐ Once a day
- ☐ Once a week

b. The shape of the pill?
- ☐ Liquid
- ☐ Oval
- ☐ Circle

c. Color of pill?

d. If you break it open, was there powder inside?
- ☐ Powder inside (capsule)
- ☐ Pill was same texture inside and out (tablet)

28. How often was your child/ren supposed to take it?
    - ☐ Once a day
    - ☐ Once a week
    - ☐ Don't know

29. Did your child/ren take it as prescribed or did you miss or skip any doses?

- ☐ No, he/she took it as prescribed
- ☐ Missed/skipped doses
  - ☐ Missed one
  - ☐ Missed a few days
  - ☐ Missed a lot of days
- ☐ He/she stopped taking it

29a. **<u>If your child/ren skipped or missed doses or stopped taking it</u>**: What were the reasons?

- ☐ Forgot to take medicine
- ☐ The pharmacy didn't give me enough
- ☐ Lost it
- ☐ Child couldn't tolerate bad taste of medicine
- ☐ Had side effects (describe): ____
- ☐ Other: ____

30. Do you remember how long were your child/ren supposed to take it after you got back?

- ☐ Stop right when I got back
- ☐ For <3 days after I got back
- ☐ 1 week after I got back
- ☐ 2 weeks after I got back
- ☐ 4 weeks after I got back
- ☐ Can't remember

31. After you got back, how long did your child/ren take it?

- ☐ Stopped right when I got back
- ☐ For <3 days after I got back
- ☐ 1 week after I got back
- ☐ 2 weeks after I got back
- ☐ 3 weeks after I got back
- ☐ 4 weeks after I got back

32. **<u>If your child/ren didn't take medications,</u>** why not?

[*Not prescribed only*:]

- ☐ No one told me I needed preventive medications
- ☐ Didn't know there were preventive medications

[*Prescribed, didn't take it only*:]

- ☐ Could not get medication at pharmacy/pharmacy did not stock medication

[*Either*:]

- ☐ It was too expensive
- ☐ Not a serious disease
- ☐ I would rather take my chances
- ☐ Don't think I needed it
- ☐ The medication has too many side effects/am worried about side effects
- ☐ I planned to just buy medicine if I got sick while traveling/easier to just treat if you get sick

- ☐ My trip was too long for me to take a prevention medication
- ☐ They didn't give me enough for my whole trip
- ☐ My insurance didn't cover it
- ☐ I forgot
- ☐ I didn't think there was malaria where I was going
- ☐ I planned to get it when I got to my destination
- ☐ It wasn't the rainy season
- ☐ Medications do not work well
- ☐ I don't like to take medications
- ☐ Other: ____

32b. Are there any other reasons that your child/ren didn't take medication? (ask question again and use options above)

Section 6: Post-travel questions

My next set of questions is about when you got back from your recent trip to _________:

33. When you started to feel sick/first noticed your symptoms, what did you think was making you sick?
    - ☐ Malaria
    - ☐ Something else (describe): ____

34. When you noticed your symptoms, where was the first place you looked for information/advice about your illness?
    - ☐ Family or friend abroad
    - ☐ Healthcare provider abroad
    - ☐ Family or friend in US
    - ☐ Primary care in US
    - ☐ Urgent care/ER in US
    - ☐ Travel clinic in US
    - ☐ Internet
    - ☐ Other: ___________________________

35. Did you try to treat yourself before seeking medical care?
    - ☐ Yes
    - ☐ No

35a. If **<u>yes</u>**, why? (*Check all that apply*):

- ☐ Doctors in the US don't know about malaria
- ☐ Going to the doctor is too expensive
- ☐ I knew it was malaria and how to treat it
- ☐ I already had the medicine
- ☐ Buying medicine is too expensive
- ☐ Didn't know it was malaria
- ☐ Malaria is easy to treat
- ☐ Malaria is not a serious problem
- ☐ Other: ____

35b. If **<u>yes</u>**, what did you do to treat yourself? (*Check all that apply*):

- ☐ Took a malaria medication. What medicine? ___________________________________
- ☐ Took medication for fever and/or pain? (e.g. Tylenol, ibuprofen, paracetamol)

If **<u>yes,</u>** what medicine: ____

- ☐ Herbal remedy: ____
- ☐ Other: ____

SKIP question 36 if the participant did not travel with a child

36. How many times did you do to the doctor before you were told you had malaria?
    - ☐ One
    - ☐ Two
    - ☐ Three
    - ☐ Four or more
    - ☐ Never told I had malaria

37. Did you have any concerns about going to the doctor with malaria symptoms?
    - ☐ Yes. Please explain: ____
    - ☐ No

38. After returning from any trip to a country with malaria, have you ever self-treated a child in your family for malaria before going to a doctor?
    - ☐ Yes – **Go to 38a.**
    - ☐ No – I did not try to self-treat the child before going to the doctor – **Go to 38b.**
    - ☐ No – Child has never seemed sick with malaria after a trip – **Go to 39**

38a. **<u>If yes,</u>** why? (*Check all that apply*):
- ☐ Malaria is easy to treat
- ☐ Malaria is not a serious problem
- ☐ Didn't know it was malaria
- ☐ I already had the medicine
- ☐ Buying medicine is too expensive
- ☐ Going to the doctor is too expensive
- ☐ I knew it was malaria and how to treat it
- ☐ Doctors in the US don't know about malaria
- ☐ Other:

36b. **<u>If no,</u>** why not? *(please explain)*: ____

Section 7: Future plans

39. Next time you travel to a country with malaria, where would you seek advice on your health?
    Check all participant mentions
    - ☐ Internet
    - ☐ Travel agent
    - ☐ Pharmacy
    - ☐ Urgent care
    - ☐ Primary care
    - ☐ Travel clinic
    - ☐ Friends / family
    - ☐ Community leaders
    - ☐ CDC / MDH
    - ☐ Other: ____________________________
    - ☐ Not going to seek advice

    Why not? ____________________________

S2.2) Consent and Survey – Final Case Interview

40. The next time you travel to a country with malaria, will you take a malaria prevention medication?
    - ☐ Yes
    - ☐ No

If no, why not?

- ☐ Too expensive
- ☐ Not a serious disease
- ☐ I would rather take my chances
- ☐ Don't think I'll need it
- ☐ I will probably forget
- ☐ I don't like to take medications
- ☐ My insurance doesn't cover it
- ☐ Medications do not work well
- ☐ It won't be the rainy season
- ☐ Can't get enough medication for my whole trip
- ☐ I don't think there is malaria where I'll travel
- ☐ I will get it when I got to my destination
- ☐ The medication has too many side effects/am worried about side effects
- ☐ I plan to just buy medicine if I get sick while traveling/easier to treat if you get sick
- ☐ My trip will be too long for me to take a prevention medication
- ☐ I can't find a pharmacy that stocks the medication
- ☐ Other: ____________________________

41. The next time you travel to a country with malaria, will you use a bed net?
    - ☐ Yes
    - ☐ No – if no, why not? ____

42. The next time you travel to a country with malaria, will you use mosquito repellent?
    - ☐ Yes
    - ☐ No – if no, why not? ____

43. Is there anything you know about malaria now that you wish you had known before you got sick?

44. In your opinion, what would be the best way to educate people about malaria?

Additional Demographic Information

45. Gender:
    - ☐ Male
    - ☐ Female
    - ☐ Other: ______________________
46. Were you born in the United States?
    - ☐ Yes
    - ☐ No

    46a. If no: Where were you born? (Country): ___________________________
    46b. If no: When did you last live there? __________________
    46c. If no: How long did you live there? __________________
    46d. If no: How long have you lived in the US? _________________
47. Have you ever lived in a country other than the United States or the country you were born?
    - ☐ Yes – where? ____
    - ☐ No

    47a. If yes: When did you last live there? __________________
    47b. If yes: How long did you live there? ______________
48. (Since living in the United States) how many times have you traveled to places with malaria / Africa?
    - ☐ 1 – this was my first time back
    - ☐ 2
    - ☐ 3
    - ☐ 4
    - ☐ 5 or more times
49. Where were your parents born? (Country): Mom: _________________ Dad: ______________________
50. How would you describe your race/ethnicity? ____
51. What is the highest level of educational you completed?

☐ Grade school ☐ Some high school ☐ High school
☐ Associate degree / some college / post-secondary training ☐ Bachelor's degree
☐ Graduate school / PhD / Advanced

52. Primary care doctors are doctors you go to for checkups and health problems that aren't emergencies. Do you go to a primary care doctor?
    - ☐ Yes
    - ☐ No

52a. If yes, when is the last time you went to your primary care doctor?
- ☐ Within the last month
- ☐ Within the last 6 months
- ☐ Within the last year
- ☐ 1-2 years
- ☐ More than 2 years ago

53. Additional notes: ____

S2.3) Consent and Survey – Emergency Department

Survey Date: ___________ Interviewer Initials: __________ Unique Participant ID: _______________
Participated? (Y/N) ___________ Completed? (Y/N)___________

Target Population:
First and second generation immigrants from Sub-Saharan Africa who have traveled to a malaria area in any part of Africa in the past 10 years, or who plan to travel in the coming 1 year

Inclusion Criteria

- ☐ **Chart:** Presenting to ED waiting room in stable status (non-critical)
- ☐ **Chart/Ask:** 18 years of age or older
- ☐ **Chart/Ask:** Place of birth in a Sub-Saharan African country: ____________________

—OR— Reported ethnicity from Sub-Saharan Africa: ____________________

- ☐ **Ask:** Traveled to a malaria-endemic area of Africa in the past 10 years or plan to travel in the next year
- ☐ **Ask:** Resides in the United States
- ☐ **Ask:** Has heard of malaria before

1. In the past 10 years, have you traveled from the US to any country in Africa?
   - ☐ Yes → When was the last time? ___________ Where did you go? ________________ →INCLUDE
   - ☐ No → Do you plan to travel to a county in Africa in the next one year?
     - ☐ Yes → Where? ________________________ → INCLUDE
     - ☐ No → DOES NOT FIT INCLUSION CRITERIA

2. Do you live in Minnesota or in the United States?
   - ☐ Yes → INCLUDE
   - ☐ No → DOES NOT FIT INCLUSION CRITERIA

3. Have you heard of malaria?
   - ☐ Yes →INCLUDE
   - ☐ No → DOES NOT FIT INCLUSION CRITERIA

---IF PATIENT FULFILLS ALL INCLUSION CRITERIA ABOVE, COMPLETE ENROLLMENT PROCESS BEFORE BEGINNING SURVEY ---

Malaria Survey – Information for Participants

We are inviting you to participate in a voluntary research survey about malaria. Malaria is a disease that exists in many tropical countries of the world. The Minnesota Department of Health (MDH) and the Hennepin County Medical Center (HCMC) are organizing this study.

The purpose is to talk about ways to prevent getting malaria when traveling in Africa. We will ask you to take part in a short survey about malaria. Please take some time to read this form or talk about this information with the research assistant.

1. WHAT IS THE REASON FOR THIS SURVEY?

This survey is research to learn about travelers and malaria. We want to learn about the experiences of people like you who visit friends and relatives in places with malaria. We hope to work with the local community, doctors, and hospitals to help people avoid getting malaria while traveling.

2. WHAT WILL I BE ASKED TO DO?

If you agree to take part in this study, we will ask you to participate in a 10-minute survey. We will ask you questions about your knowledge of malaria and attitudes towards malaria. The research assistant will write down your responses on the computer or paper survey form. Your identity or answers will not be shared with anyone outside the study team.

Participating in this survey will not delay you from seeing a doctor.

3. IS PARTICIPATION VOLUNTARY?

Yes. Your participation in this survey is voluntary. You have the right to not answer any questions if you don't want to. You also have the right to stop participating in the survey at any time. There are no penalties or consequences if you decide that you do not want to participate.

Your relationship with HCMC, MDH, and their staff/employees will not be affected or harmed if you decided you do not want to participate in this survey.

4. WHAT IF I HAVE QUESTIONS?

We will be happy to answer any questions you have about this study. If you have concerns or questions about this study, please contact:
Hannah Volkman, Survey Coordinator at 651-201-5414 or hannah.volkman@state.mn.us

For questions about your rights as a participant in this study, contact the Hennepin County Medical Center Institutional Review Board, at 612-863-6882.

***** Note to interviewer: unless instructed to do so below, do <u>not</u> read answers to respondents. *****

Section 1: Malaria Knowledge

I want to understand what you know about malaria. I'll read you a few statements and ask you to rate them on a scale of 1 to 5:
1 – Not true **3 – Somewhat true** **5 – Very true/always true**

1. How true are the following statements?

   1a. I am concerned for myself about getting malaria when traveling

   1 2 3 4 5

   1b. Malaria can be a deadly disease

   1 2 3 4 5

2. When traveling to areas with malaria, who is more likely to become sick from malaria – you or your child?
   - ☐ You / Adult
   - ☐ Child
   - ☐ Equally likely

3. How do people get malaria? (*check all that are mentioned and write in others*)
   - ☐ From mosquitoes
   - ☐ From bad air
   - ☐ From drinking water
   - ☐ From another person ***<u>(prompt with clarifier #4)</u>***
   - ☐ Other (please describe): ____________________

4. Can people catch malaria directly from another person who is sick with malaria?
   Directly means spreading from one person right to another.
   - ☐ Yes
   - ☐ No

5. Can you prevent malaria?
   - ☐ Yes

5a. **<u>If yes</u>**, how can you prevent malaria? *(check all that the participant lists)*
   - ☐ Immunizations/shots
   - ☐ Bed nets
   - ☐ Insect repellent
   - ☐ Taking medications
   - ☐ Other (describe): ____________________

- ☐ No

Section 2: Previous Malaria Exposure and Infection

6. Have you ever had malaria?
   - ☐ Yes
   - ☐ No
   - ☐ Don't know

Section 3: Most Recent Trip / Upcoming Trip

*****Only use <u>upcoming</u> trip prompts if participant has <u>not</u> traveled to a malaria area in the past 10 years*****

Now I'd like to find out more about your **most recent trip to ______** / the trip you plan to take to ____________

7. What were the main reasons for your travel? / What are the main reasons you are going to travel?
   If more than one rank primary (#1), secondary reasons (#2), etc.

_____ Visit friends or relatives
_____ Business
_____ Vacation – not visiting friends or relatives
_____ Studying abroad / research
_____ Mission trip
_____ Other: ____

8. What country(ies) did you travel to? / What country(ies) will you travel to? ___________________________

9. How long were you on your trip? / How long will you be on your trip? (indicate weeks/days) _____________

10. Did you talk to someone about malaria before you left? / Will you talk with someone about malaria before you go?

   ☐ Yes **– Go to question 10a**
   ☐ No **– Skip to question 11**

10a. **<u>If yes</u>**, who did you talk to? / Who will you talk to?

   ☐ Family member / friend
   ☐ Community elder
   ☐ Healthcare provider
   (check 11 as Yes, skip to 11a)
   ☐ Religious leader
   ☐ Travel agent
   ☐ Other: ____________________________

11. ***If participant <u>does not</u> list healthcare provider above:*** Did you see a healthcare provider before you left? / Do you plan to see a healthcare provider before you go?
   ☐ **Yes – Go to question 11a**
   ☐ **No – Why not?** ____

11a. **<u>If yes</u>*:*** Was / Is it your regular healthcare provider, travel clinic, or somewhere else?

   ☐ Regular healthcare provider (primary care)
   ☐ Travel clinic
   ☐ Both regular healthcare provider and travel clinic
   ☐ Urgent care
   ☐ Pharmacy / minute clinic
   ☐ Other: ____

12. If you traveled as a family, was everyone traveling seen by a healthcare provider?
   ☐ Yes
   ☐ No
   ☐ Some were seen → How are the people that were seen related to you (e.g., son, mother, etc.)?
   ____

12a. Were you more concerned about making sure certain family members were seen by a healthcare provider than others?

- ☐ No
- ☐ Yes – Why? (check all that apply)
  - ☐ Only certain family members covered by insurance
  - ☐ Cost
  - ☐ Risk of illness
  - ☐ Age (very young or very old)
  - ☐ Pregnancy
  - ☐ Other: ____

13. During your most recent trip did you use any of the following strategies to keep mosquitos away or stay healthy? / Do you plan to use any of the following strategies to keep mosquitos away or stay healthy?

I'm going to list a few examples and you can respond: yes, no, or sometimes

☐ Yes	☐ No	☐ Sometimes	Bed nets
☐ Yes	☐ No	☐ Sometimes	Repellent / creams / sprays / lotions
☐ Yes	☐ No	☐ Sometimes	Mosquito coils
☐ Yes	☐ No	☐ Sometimes	Environmental cleanup i.e., removing sources of standing water
☐ Yes	☐ No	☐ Sometimes	Staying indoors when mosquitoes are out
☐ Yes	☐ No	☐ Sometimes	Wear long clothing
☐ Yes	☐ No	☐ Sometimes	Practice good food safety / hygiene
☐ Yes	☐ No	☐ Sometimes	Ensure clean water source (safe, bottled)
☐ Yes	☐ No	☐ Sometimes	Educate yourself on risks of malaria before traveling
☐ Yes	☐ No	☐ Sometimes	Pick where you stayed in order to avoid mosquitos (AC/screens)
☐ Yes	☐ No	☐ Sometimes	Get vaccinations

Anything else? ____

*****If participant is a prospective traveler, SKIP to question 22 *****

Section 4: Antimalarial Use During Travel

14. Did you take a malaria prevention medicine during your last trip?

- ☐ Yes → Did you finish taking the medicine after you got back? ☐ Yes ☐ No ☐ Don't recall
- ☐ No
- ☐ Sometimes → Did you finish taking the medicine after you got back? ☐ Yes ☐ No ☐ Don't recall

15. Did a healthcare provider write you a prescription for a malaria prevention medicine?

- ☐ Yes
- ☐ No

15a**. <u>If yes,</u>** did you pick up the medicine in the US or once you arrived to your destination?

- ☐ Picked up the medicine in the US
- ☐ Picked up the medicine in destination country

If 14 is yes/sometimes and 15 is no, clarify: Did you buy the malaria prevention medicine without a prescription once you arrived to your destination?

- ☐ Yes
- ☐ No – Explain: ____

Section 5: Traveling with Children

16. On your last trip did any children travel with you?
    - ☐ Yes – if yes, how many? ___________________ How old were they? ________________________
    - ☐ No

16a. **<u>If yes</u>**, did the child/ren take a malaria prevention medicine during your last trip?
- ☐ Yes → Did the child finish taking the medicine after he/she got back? ☐ Yes ☐ No ☐ Don't recall
- ☐ No
- ☐ Sometimes→Did the child finish taking the medicine after he/she got back? ☐ Yes ☐ No ☐ Don't recall

16b. Did a healthcare provider write your child/children a prescription for malaria prevention medicine?
- ☐ Yes
- ☐ No

16c. **<u>If yes</u>,** did you pick up the medicine for your child/ren in the US or once you arrived to your destination?
- ☐ Picked up the medicine in the US
- ☐ Picked up the medicine in destination country

<u>If 16a is yes/sometimes and 16b is no</u>: Did you buy the malaria prevention medicine for your child/ren without a prescription once you arrived to your destination?
- ☐ Yes
- ☐ No Explain: ____

Section 6: Illnesses During Travel and Healthcare Seeking Behaviors

17. Since living in the United States, do you think you have gotten malaria while traveling outside of the US?
    - ☐ Yes
    - ☐ No – **Skip to question 22**

18. When you noticed your symptoms, where was the first place you looked for information/advice about your illness?
    - ☐ Family or friend abroad
    - ☐ Healthcare provider abroad
    - ☐ Family or friend in US
    - ☐ Primary care in US
    - ☐ Urgent care/ER in US
    - ☐ Travel clinic in US
    - ☐ Internet
    - ☐ Other: _____________________________

19. Did you try to treat yourself before seeking medical care?
    - ☐ Yes
    - ☐ No

19a. **<u>If yes</u>**, why? (*Check all that apply*):
- ☐ Doctors in the US don't know about malaria
- ☐ Going to the doctor is too expensive
- ☐ I knew it was malaria and how to treat it
- ☐ I already had the medicine
- ☐ Buying medicine is too expensive
- ☐ Didn't know it was malaria
- ☐ Malaria is easy to treat
- ☐ Malaria is not a serious problem
- ☐ Other: ____

19b. **If yes**, what did you do to treat yourself? (*Check all that apply*):

- ☐ Took malaria medication. Which medicine? ___________________________________
- ☐ Took medication for fever and/or pain? (e.g., Tylenol, ibuprofen, paracetamol)

If yes, which medicine: ____

- ☐ Herbal remedy: ____
- ☐ Other: ____

20. Did you end up seeing a healthcare provider when you thought you had malaria?

☐ **Yes (answer questions 20a through 20d)**

20a. What made you decide to go? ____

20b. Were you tested for malaria?

☐ Yes ☐ No ☐ Don't know / don't remember

20c. Did a healthcare provider tell you that you had malaria?

☐ Yes ☐ No ☐ Don't know / don't remember

20d. How many times did you see a healthcare provider before you were told you had malaria?

☐ One ☐ Two ☐ Three ☐ Four or more ☐ Don't remember

☐ **No** 20f. **If no:** Why not?

- ☐ I got better on my own
- ☐ Malaria is easy to treat
- ☐ Malaria is not a serious problem
- ☐ I already had the medicine
- ☐ Buying medicine is too expensive
- ☐ Going to the doctor is too expensive
- ☐ I knew it was malaria and how to treat it
- ☐ Doctors in the US don't know about malaria
- ☐ Other: _________________________________

21. After returning from any trip to a country with malaria, have you ever self-treated a child in your family for malaria before seeing a healthcare provider?

- ☐ Yes – **Go to question 21a**
- ☐ No – I did not try to self-treat the child before going to the doctor - **Skip to question 21b**
- ☐ No – Child never seemed sick with malaria – **Skip to question 22.**

21a. **If yes,** why? (*Check all that apply*):

- ☐ Malaria is easy to treat
- ☐ Malaria is not a serious problem
- ☐ Didn't know it was malaria
- ☐ I already had the medicine
- ☐ Buying medicine is too expensive
- ☐ Going to the doctor is too expensive
- ☐ I knew it was malaria and how to treat it
- ☐ Doctors in the US don't know about malaria
- ☐ Other: _________________________________

21b. **If no,** why not? *(please explain)*: ____

22. Imagine you just got back to the United States from a trip to [country of travel] and started to feel symptoms of malaria such as fever and chills. Would you go to the doctor right away or try to treat it yourself at home first?

- ☐ I would go to the doctor right away. Why?
  - ☐ Malaria is dangerous/serious
  - ☐ Doctors can prescribe antimalarial medicines
  - ☐ Other: ____________________
- ☐ I would try to treat it at home first. Why?
  - ☐ Malaria is easy to treat; I know how to treat it
  - ☐ Malaria is not a serious problem
  - ☐ Going to the doctor is too expensive
  - ☐ Doctors in the US don't know about malaria
  - ☐ Doctors in the US would treat me bad / fear of being quarantined
  - ☐ Other: ____________________

23. Do you have any concerns about seeing a healthcare provider if you had malaria symptoms?

- ☐ Yes. Please explain: ____________________
- ☐ No

Section 7: Future Plans

24. If you traveled again to [country], where would you seek advice on your health?

Check all participant mentions

- ☐ Internet
- ☐ Travel agent
- ☐ Pharmacy
- ☐ Urgent care
- ☐ Primary care
- ☐ Travel clinic
- ☐ Friends / family
- ☐ Community leaders
- ☐ CDC / MDH
- ☐ Other: ____________________
- ☐ Not going to seek advice

Why not? ____________________

25. The next time you travel to [country], will you take a malaria prevention medication?

- ☐ Yes
- ☐ No

<u>If no</u>, why not?

- ☐ Too expensive
- ☐ Not a serious disease
- ☐ I would rather take my chances
- ☐ Don't think I'll need it
- ☐ I will probably forget
- ☐ I don't like to take medications
- ☐ My insurance doesn't cover it
- ☐ Medications do not work well
- ☐ It won't be the rainy season
- ☐ Can't get enough medication for my whole trip
- ☐ I don't think there is malaria where I'll travel
- ☐ I will get it when I got to my destination
- ☐ The medication has too many side effects/am worried about side effects
- ☐ I plan to just buy medicine if I get sick while traveling/easier to treat if you get sick
- ☐ My trip will be too long for me to take a prevention medication
- ☐ I can't find a pharmacy that stocks the medication
- ☐ Other: ____________________

26. In your opinion, what would be the best way to educate people about malaria?

____

Additional Demographic Information

27. Gender
    - ☐ Male
    - ☐ Female
    - ☐ Other: ________________________

28. Age: _____________
If older than 89 years, list ">89"

29. Were you born in the United States?
    - ☐ Yes
    - ☐ No

29a. **If no**: How long have you lived in the US? _________________
29b. **If no**: Where were you born? (Country): ___________________________
29c. **If no**: When did you last live there? __________________
29d. **If no**: How long did you live there? __________________

30. Have you ever lived in a country other than the United States or the country you were born?
    - ☐ Yes – where? ____
    - ☐ No

30a. **If yes:** When did you last live there? __________________
30b. **If yes:** How long did you live there? ______________

31. Since living in the United States, how many times have you traveled to places with malaria/ Africa?
    - ☐ Never – this will be my first time
    - ☐ 1
    - ☐ 2
    - ☐ 3
    - ☐ 4
    - ☐ 5 or more times

32. Where were your parents born? (Country): Mom: _________________ Dad: ______________________

33. How would you describe your race/ethnicity? ____

34. What is the highest level of education you completed?
    - ☐ Grade school
    - ☐ Some high school
    - ☐ High school
    - ☐ Associate degree / some college / post-secondary training
    - ☐ Bachelor's degree
    - ☐ Graduate school

35. Primary care doctors are doctors you go to for checkups and health problems that aren't emergencies. Do you go to a primary care doctor?
    - ☐ Yes
    - ☐ No

35a. If yes, when is the last time you went to your primary care doctor?
- ☐ Within the last month
- ☐ Within the last 6 months
- ☐ Within the last year
- ☐ 1-2 years
- ☐ More than 2 years ago

36. Additional notes: ____

S2.4) Consent and Survey – Community Paper and Online

Malaria Survey - Information for Participants

We are inviting you to complete a short survey about malaria. Malaria is a disease that exists in many tropical countries of the world. The Minnesota Department of Health (MDH) is organizing this study.
Please take some time to read this form or talk about this information with the research assistant.

1. WHAT IS THE REASON FOR THIS SURVEY?

This survey is research to learn about travelers and malaria. We want to learn about the experiences of people like you who visit friends and relatives in places with malaria. We hope to work with the local community, doctors, and hospitals to help people avoid getting malaria while traveling.

2. WHAT WILL I BE ASKED TO DO?

If you agree to take part in this study, we will ask you to participate in a 10-minute survey. We will ask you questions about your knowledge of malaria and attitudes towards malaria. You will enter your responses into the survey. If the survey is being done with a research assistant, he/she will write down your responses on the computer or paper survey form. Your identity or answers will not be shared with anyone outside the study team.

3. IS PARTICIPATION VOLUNTARY?

Yes. Your participation in this survey is voluntary. You have the right to not answer any questions if you don't want to. You also have the right to stop participating in the survey at any time. There are no penalties or consequences if you decide that you do not want to participate. Your relationship with MDH, and their staff/employees will not be affected or harmed if you decided you do not want to participate in this survey.

4. WHAT IF I HAVE QUESTIONS?

We will be happy to answer any questions you have about this study. If you have concerns or questions about this study, please contact: Hannah Volkman, Survey Coordinator at 651-201-5414 or hannah.volkman@state.mn.us For questions about your rights as a participant in this study, contact the Minnesota Department of Health Institutional Review Board, at 651-201-5942. You may download a copy of the information provided on this page if you would like to refer back to this information.

☐ **I understand participation in the survey is voluntary and consent to participate**

S2.4) Consent and Survey – Community Paper and Online

INCLUSION CRITERIA

Please return this page to the staff member to review to see if you are eligible to participate.

1. Are you 18 years of age or older?
   - ☐ Yes
   - ☐ No

2. Were you born in a Sub-Saharan African country?
   - ☐ Yes. What country? ____________________
   - ☐ No

   Were your parents born in a Sub-Saharan African country?
   - ☐ Yes.
     Where was your mom born? ______________________
     Where was your dad born? _______________________
   - ☐ No

3. In the past 10 years, have you traveled from the US to any country in Africa?
   - ☐ Yes
     When was the last time? _________________
     Where did you go? _________________
   - ☐ No
     Do you plan to travel to a county in Africa in the next one year?
     - ☐ Yes
       Where? _________________________
     - ☐ No

4. Do you live in the United States?
   - ☐ Yes
   - ☐ No

5. Have you heard of malaria?
   - ☐ Yes
   - ☐ No

IF you have traveled in the past please answer all questions about your most recent trip

Please rate the following statements on a scale of 1 to 5:
1 – Not true **3 – Somewhat true** **5 – Very true/always true**

1. How true are the following statements?

   1a. I am concerned for myself about getting malaria when traveling

   1 2 3 4 5

   1b. Malaria can be a deadly disease

   1 2 3 4 5

2. **How do people get malaria?** *You may list multiple ways.*

   ____

3. **Can you prevent malaria?**

☐ Yes → How can you prevent malaria? ____
☐ No

4. **Have you ever had malaria?**

☐ Yes
☐ No
☐ I don't know

S2.4) Consent and Survey – Community Paper and Online

Now I'd like to find out more about your **most recent past trip** / the trip you plan to take

5. What were the main reasons for your travel? / What are the main reasons you are going to travel?
   If more than one rank primary (#1), secondary reasons (#2), etc.
   _____ Visit friends or relatives
   _____ Business
   _____ Vacation – not visiting friends or relatives
   _____ Studying abroad / research
   _____ Mission trip
   _____ Other: ____

6. What country(ies) did you travel to? / What country(ies) will you travel to?

____

7. How long were you on your trip? / How long will you be on your trip? (indicate weeks)

____

8. Did you talk to someone about avoiding malaria before you left? / Will you talk with someone about avoiding malaria before you go?

   ☐ Yes
   Who did you talk to? / Who will you talk to? ____
   ☐ No

9. Did you see a healthcare provider before you left? / Do you plan to see a healthcare provider before you go?
   ☐ Yes → Was / Is it your regular healthcare provider, travel clinic, or somewhere else?
      ☐ Regular healthcare provider (primary care)
      ☐ Travel clinic
      ☐ Both regular healthcare provider and travel clinic
      ☐ Urgent care
      ☐ Pharmacy / minute clinic
      ☐ Other: ____

   ☐ No – Why not? ____

10. During your most recent trip did you use any of the following strategies to keep mosquitos away or stay healthy? / Do you plan to use any of the following strategies to keep mosquitos away or stay healthy?

☐ Yes ☐ No — Bed nets

☐ Yes ☐ No — Repellent / creams / sprays / lotions

☐ Yes ☐ No — Mosquito coils

☐ Yes ☐ No — Staying indoors when mosquitoes are out

☐ Yes ☐ No — Wear long clothing

☐ Yes ☐ No — Educate yourself on risks of malaria before traveling

☐ Yes ☐ No — Pick where you stayed in order to avoid mosquitos (Air conditioning/screens)

11. Did you take a malaria prevention medicine during your last trip?

☐ Yes → Did you finish taking the medicine after you got back? ☐ Yes ☐ No ☐ Don't recall

☐ No

12. Since living in the United States, do you think you have gotten malaria while traveling outside of the US?

☐ Yes

*Answer 12a-12d on the **last** time you thought you had malaria while traveling outside the US:*
Did you end up seeing a healthcare provider when you thought you had malaria?

☐ **Yes**

12a. What made you decide to seek care? ____

12b. Were you tested for malaria?

☐Yes ☐No ☐Don't know / don't remember

12c. Did a healthcare provider tell you that you had malaria?

☐Yes ☐No ☐ Don't know / don't remember

12d. How many times did you see a healthcare provider before you were told you had malaria?

☐ One ☐Two ☐ Three ☐Four or more ☐ Don't remember

No → Why not? ____

12f. Did you try to treat yourself?

☐ Yes

☐ No

12g. Anything else to share about the last time you got malaria while traveling outside the US?

____

☐ No

13. Imagine you just got back to the United States from a trip to [country of travel] and started to feel symptoms of malaria such as fever and chills. Would you go to the doctor right away or try to treat it yourself at home first?

☐ I would go to the doctor right away. Why? ____

☐ I would try to treat it at home first. Why? ____

14. Do you have any concerns about seeing a healthcare provider if you had malaria symptoms?
- ☐ Yes. Please explain: ____
- ☐ No

15. If you traveled again to [country], where would you seek advice on your health before you leave?

____

16. The next time you travel to [country], will you take a malaria prevention medication if recommended?
- ☐ Yes
- ☐ No
  Why not?____

17. In your opinion, what would be the best way to educate people about malaria?

____

18. Gender
    - ☐ Male
    - ☐ Female
    - ☐ Other: ___________________________

19. Age: ______________

20. Were you born in the United States?
    - ☐ Yes
    - ☐ No

    20a. **<u>If no</u>**: How long have you lived in the US? ___________________

    20b. **<u>If no</u>**: Where were you born? (Country): ___________________

    20c. **<u>If no</u>**: When did you last live there? ____________________

    20d. **<u>If no</u>**: How long did you live there? ____________________

21. Since living in the United States, how many times have you traveled to places with malaria/ Africa?
    - ☐ Never – this will be my first time
    - ☐ 1
    - ☐ 2
    - ☐ 3
    - ☐ 4
    - ☐ 5 or more times

22. Where were your parents born? (Country): Mom: ______________ Dad: ____________________

23. What is the highest level of education you completed?
    - ☐ Grade school
    - ☐ Some high school
    - ☐ High school
    - ☐ Associate degree / some college / post-secondary training
    - ☐ Bachelor's degree
    - ☐ Graduate school

THANK YOU FOR COMPLETING THE SURVEY!!

Target Population:
All prospective travelers to Africa visiting a HealthPartners travel clinic that speak one of the four survey languages.

Survey Languages:

English
French
Somali
Amharic

Inclusion Criteria

- ☐ **Chart:** Presenting to a HealthPartners travel clinic in stable condition (non-critical)
- ☐ **Chart:** 18 years of age or older
- ☐ **Chart:** Traveling to Africa in the next 1 year (from clinic visit)
- ☐ **Ask:** Resides in the United States
- ☐ **Ask:** Has heard of malaria before

1. Have you heard of malaria? **(Record "No" responses)**
   - ☐ Yes →INCLUDE
   - ☐ No → DOES NOT FIT INCLUSION CRITERIA

2. Do you live in Minnesota or in the United States? **(Record "No" responses)**
   - ☐ Yes → INCLUDE
   - ☐ No → DOES NOT FIT INCLUSION CRITERIA

---IF PATIENT FULFILLS ALL INCLUSION CRITERIA ABOVE, COMPLETE ENROLLMENT PROCESS BEFORE BEGINNING SURVEY ---

INFORMATION AND CONSENT FORM

Program Title: Reducing U.S. Malaria by Testing Multiple Interventions

HealthPartners Investigator:
Pat Walker, MD, MPH
HealthPartners Institute
8170 33rd Ave S
Mail Stop 23301A
Bloomington, MN 55425-1524
Telephone Number: 952-967-7956

We are inviting you to participate in a research study about malaria. Malaria is a disease that exists in many tropical countries of the world. HealthPartners is working with the Minnesota Department of Health (MDH) on this study.

1. WHAT IS THE PURPOSE OF THIS STUDY?
The purpose of this study is to talk about ways to prevent getting malaria when traveling in Africa.
We want to learn about the experiences of people like you who visit friends and relatives in places with malaria. We hope to work with the local community, doctors, and hospitals to help people avoid getting malaria while traveling.

2. WHO IS SPONSORING THE STUDY?
HealthPartners Institute is receiving financial support from MDH ("the Sponsor") to assist in conducting this research study. The amount of payment is enough to cover the study doctor's and/or institution's expenses to perform the study.

3. WHAT WILL I BE ASKED TO DO?
If you agree to take part in this study, we will ask you to participate in a 20-minute survey. We will ask you questions about your knowledge of malaria and your thoughts towards malaria. The research assistant will write down your responses on the computer or paper survey form. Your identity or answers will not be shared with anyone outside the study team.

We will also ask you to participate in a 5-minute phone call survey after you return to the United States.

4. ARE THERE ANY RISKS OR BENEFITS IN PARTICIPATING?
There are no anticipated risks to you for participating in the survey. Study subjects may experience some emotional discomfort from recalling experiences with malaria. The research assistants will make a conscious effort to reduce potential risks. You may or may not benefit from this study.

5. IS PARTICIPATION VOLUNTARY?
Yes. Your participation in this survey is voluntary. Your decision whether or not to partake in this study is completely voluntary and will not affect your current, or future relations, with HealthPartners or any of its providers. You have the right to not answer any questions if you don't want to. You also have the right to stop participating in the survey at any time.

The information you provide us for this study will be kept confidential and you will never be identified in any report or publication in regards to this study. At the end of the study, all forms with your name or other identifying information will be destroyed.

6. Will I be paid to participate?
You will be paid a $10 Target gift card for participating today. We may have to report this payment to the IRS, but your identity or answers will not be shared with the IRS.

7. WHAT IF I HAVE QUESTIONS?

We will be happy to answer any questions you have about this study. If you have concerns or questions about this study, please contact: Alison Helm at 952-883-6076 or alison.m.helm@healthpartners.com

For questions about your rights as a participant in this study, you may call the Office of Research Subjects at 952-967-5268.

Version 12.26.2017

***** Note to interviewer: unless instructed to do so below, do not read answers to respondents. *****

Section 1: Malaria Knowledge

I want to understand what you know about malaria. I'll read you a few statements and ask you to rate them on a scale of 1 to 5:

1 – Not true **3 – Somewhat true** **5 – Very true/always true**

1. How true are the following statements?

   1a. I am concerned for myself about getting malaria when traveling

   1 2 3 4 5

   1b. Malaria can be a deadly disease

   1 2 3 4 5

2. When traveling to areas with malaria, who is more likely to become sick from malaria – you or your child?
   - ☐ You / Adult
   - ☐ Child
   - ☐ Equally likely

3. How do people get malaria? (*check all that are mentioned and write in others*)
   - ☐ From mosquitoes
   - ☐ From bad air
   - ☐ From drinking water
   - ☐ From another person ***(prompt with clarifier #4)***
   - ☐ Other (please describe):____

4. Can people catch malaria directly from another person who is sick with malaria?
   Direct means spreading from one person right to another.
   - ☐ Yes
   - ☐ No

5. Can you prevent malaria?
   - ☐ Yes

5a. **If yes**, how can you prevent malaria? *(check all that the participant lists)*
   - ☐ Immunizations/shots
   - ☐ Bed nets
   - ☐ Insect repellent
   - ☐ Take medications
   - ☐ Other (describe): ____

   - ☐ No

Section 2: Previous Malaria Exposure and Infection

6. Have you ever had malaria?
   - ☐ Yes
   - ☐ No
   - ☐ Don't know

Section 3: Upcoming Trip

Now I'd like to find out more about your upcoming trip

7. Where will you travel to? *(List specific country[ies])* ______________________

8. How long will you be on your trip? If unknown, approximate: ________________________________

9. What are the main reasons you are going to travel?
   If more than one rank primary (#1), secondary reasons (#2), etc.

______ Visit friends or relatives
______ Business
______ Vacation – not visiting friends or relatives
______ Studying abroad / research
______ Mission trip
______ Other: ____

10. Do you plan to use any of the following strategies to keep mosquitos away or stay healthy?
I'm going to list a few examples and you can respond: yes, no, or sometimes

- ☐ Yes ☐ No Bed nets
- ☐ Yes ☐ No Repellent / creams / sprays / lotions
- ☐ Yes ☐ No Mosquito coils
- ☐ Yes ☐ No Environmental cleanup i.e., removing sources of standing water
- ☐ Yes ☐ No Staying indoors when mosquitoes are out
- ☐ Yes ☐ No Wear long clothing
- ☐ Yes ☐ No Practice good food safety / hygiene
- ☐ Yes ☐ No Ensure clean water source (safe, bottled)
- ☐ Yes ☐ No Educate yourself on risks of malaria before traveling
- ☐ Yes ☐ No Pick where you stay in order to avoid mosquitos (AC/screens)
- ☐ Yes ☐ No Get vaccinations

Anything else? ____

11. Did someone tell you to come to this travel clinic?

- ☐ Yes, Who?
  - ☐ Primary care provider / family physician / "regular" doctor
  - ☐ Friend or family member
  - ☐ Insurance provider
  - ☐ Other: ______________________________
- ☐ No, I chose to come to the travel clinic on my own

12. Why did you choose to visit the travel clinic before your trip?

- ☐ Need immunization required for travel
- ☐ Generally, want to prevent illnesses while traveling
- ☐ Specifically, want to prevent malaria while traveling
- ☐ Referral from question 11
- ☐ Other: ____

13. Are you more concerned about making sure certain family members are seen by a healthcare provider than others?
    - ☐ No
    - ☐ Yes → Why? (check all that apply)
        - ☐ Only certain family members covered by insurance
        - ☐ Cost
        - ☐ Risk of illness
        - ☐ Age (very young or very old
        - ☐ Pregnancy
        - ☐ Other: ____

*****If participant is traveling for the first time, SKIP to question 23*****

Section 4: Previous Travel and Antimalarial Use

14. Have you traveled from the United States to Africa before?
    - ☐ Yes
    - ☐ No, this will be my first time traveling from the US to Africa

15. Did you take a malaria prevention medicine during your last trip?
    - ☐ Yes → Did you finish taking the medicine after you got back? ☐ Yes ☐ No ☐ Don't recall
    - ☐ No
    - ☐ Sometimes → Did you finish taking the medicine after you got back? ☐ Yes ☐ No ☐ Don't recall

16. Did a healthcare provider write you a prescription for a malaria prevention medicine?
    - ☐ Yes
    - ☐ No

16a. **<u>If yes,</u>** did you pick up the medicine in the US or once you arrived to your destination?
- ☐ Picked up the medicine in the US
- ☐ Picked up the medicine in destination country

If 15 is yes/sometimes and 16 is no, clarify: Did you buy the malaria prevention medicine without a prescription once you arrived to your destination?
- ☐ Yes
- ☐ No – Explain:

____

Section 5: Traveling with Children

17. On your last trip did any children travel with you?
    - ☐ Yes – if yes, how many? __________________ How old were they? _______________________
    - ☐ No **Skip to question 18**

17a. **<u>If yes</u>**, did the child/ren take a malaria prevention medicine during your last trip?
- ☐ Yes → Did the child finish taking the medicine after he/she got back? ☐ Yes ☐ No ☐ Don't recall
- ☐ No
- ☐ Sometimes→Did the child finish taking the medicine after he/she got back? ☐ Yes ☐ No ☐ Don't recall

17b. Did a healthcare provider write your child/children a prescription for malaria prevention medicine?

- ☐ Yes
- ☐ No

17c**.** **<u>If yes</u>,** did you pick up the medicine for your child/ren in the US or once you arrived to your destination?

- ☐ Picked up the medicine in the US
- ☐ Picked up the medicine in destination country

<u>If 17a is yes/sometimes and 17b is no</u>: Did you buy the malaria prevention medicine for your child/ren without a prescription once you arrived to your destination?

- ☐ Yes
- ☐ No Explain: ____

Section 6: Illnesses During Travel and Healthcare Seeking Behaviors

18. Since living in the United States, do you think you have gotten malaria while traveling outside of the US?
    - ☐ Yes
    - ☐ No – **Skip to question 23**

19. When you noticed your symptoms, where was the first place you looked for information/advice about your illness?
    - ☐ Family or friend abroad
    - ☐ Healthcare provider abroad
    - ☐ Family or friend in US
    - ☐ Primary care in US
    - ☐ Urgent care/ER in US
    - ☐ Travel clinic in US
    - ☐ Internet
    - ☐ Other: ___________________________

20. Did you try to treat yourself before seeking medical care?
    - ☐ Yes
    - ☐ No

20a. **<u>If yes</u>**, why? (*Check all that apply*):

- ☐ Doctors in the US don't know about malaria
- ☐ Going to the doctor is too expensive
- ☐ I knew it was malaria and how to treat it
- ☐ I already had the medicine
- ☐ Buying medicine is too expensive
- ☐ Didn't know it was malaria
- ☐ Malaria is easy to treat
- ☐ Malaria is not a serious problem
- ☐ Other: ____

20b. **<u>If yes</u>**, what did you do to treat yourself? (*Check all that apply*):

- ☐ Took malaria medication. What medicine? ____________________________
- ☐ Took medication for fever and/or pain? (e.g. Tylenol, ibuprofen, paracetamol)

<u>If yes,</u> what medicine: __________________________________

- ☐ Herbal remedy: ____
- ☐ Other: ______________________________

21. Did you end up seeing a healthcare provider when you thought you had malaria?
    - ☐ **Yes (answer questions 21a through 21d)**

21a. What made you decide to go? ____________________________________

21b. Were you tested for malaria?
☐Yes ☐No ☐ Don't know / don't remember

21c. Did a healthcare provider tell you that you had malaria?
☐Yes ☐No ☐ Don't know / don't remember

21d. How many times did you see a healthcare provider before you were told you had malaria?
☐ One ☐Two ☐ Three ☐ Four or more ☐ Don't remember

☐ **No**, 21f. **If no:** Why not?
- ☐ I got better on my own
- ☐ Malaria is easy to treat
- ☐ Malaria is not a serious problem
- ☐ I already had the medicine
- ☐ Buying medicine is too expensive
- ☐ Going to the doctor is too expensive
- ☐ I knew it was malaria and how to treat it
- ☐ Doctors in the US don't know about malaria
- ☐ Other: _________________________________

22. After returning from any trip to a country with malaria, have you ever self-treated a child in your family for malaria before or instead of going to a doctor?
- ☐ Yes – **Go to 22a.**
- ☐ No – I did not try to self-treat the child before going to the doctor - **Go to 22b.**
- ☐ No – Child has never seemed sick with malaria – **Go to 23.**

22a. **If yes,** why? (*Check all that apply*):
- ☐ Malaria is easy to treat
- ☐ Malaria is not a serious problem
- ☐ Didn't know it was malaria
- ☐ I already had the medicine
- ☐ Buying medicine is too expensive
- ☐ Going to the doctor is too expensive
- ☐ I knew it was malaria and how to treat it
- ☐ Doctors in the US don't know about malaria
- ☐ Other: _________________________________

22b. **If no,** why not? *(please explain)*: ____

23. Imagine you just got back to the United States from a trip to [country of travel] and started to feel symptoms of malaria such as fever and chills. Would you go to the doctor right away or try to treat it yourself at home first?

☐ I would go to the doctor right away. Why?
- ☐ Malaria is dangerous/serious
- ☐ Doctors can prescribe antimalarial medicines
- ☐ Other: ____

☐ I would try to treat it at home first. Why?
- ☐ Malaria is easy to treat; I know how to treat it
- ☐ Malaria is not a serious problem
- ☐ Going to the doctor is too expensive
- ☐ Doctors in the US don't know about malaria
- ☐ Doctors in the US would treat me bad / fear of being quarantined
- ☐ Other: ____

24. Do you have any concerns about seeing a healthcare provider if you had malaria symptoms?
    - ☐ Yes. Please explain: ____________________
    - ☐ No

Section 7: Future Plans

25. Besides this travel clinic visit, where do you plan to seek advice on your health for this trip?
    Check all participant mentions
    - ☐ Internet
    - ☐ Travel agent
    - ☐ Pharmacy
    - ☐ Urgent care
    - ☐ Primary care
    - ☐ Travel clinic
    - ☐ Friends / family
    - ☐ Community leaders
    - ☐ CDC / MDH
    - ☐ Other: ____________________
    - ☐ Not going to seek advice

    Why not? ____________________

26. Do you plan to take a medication to prevent malaria if the doctor prescribes it for your upcoming trip?
    - ☐ Yes
    - ☐ No

<u>If no</u>, why not?
- ☐ Too expensive
- ☐ Not a serious disease
- ☐ I would rather take my chances
- ☐ Don't think I'll need it
- ☐ I will probably forget
- ☐ I don't like to take medications
- ☐ My insurance doesn't cover it
- ☐ Medications do not work well
- ☐ It won't be the rainy season
- ☐ Can't get enough medication for my whole trip
- ☐ I don't think there is malaria where I'll travel
- ☐ I will get it when I got to my destination
- ☐ The medication has too many side effects/am worried about side effects
- ☐ I plan to just buy medicine if I get sick while traveling/easier to treat if you get sick
- ☐ My trip will be too long for me to take a prevention medication
- ☐ I can't find a pharmacy that stocks the medication
- ☐ Other: ____________________

27. In your opinion, what would be the best way to educate people about malaria?

____

Additional Demographic Information

28. Gender
    - ☐ Male
    - ☐ Female
    - ☐ Other: ________________________

29. Age: ____________
    If older than 89 years, list “>89”

30. Were you born in the United States?
    - ☐ Yes
    - ☐ No

30a. **If no**: How long have you lived in the US? _________________
30b. **If no**: Where were you born? (Country): ___________________________
30c. **If no**: When did you last live there? __________________
30d. **If no**: How long did you live there? __________________

31. Have you ever lived in a country other than the United States or the country you were born?
    - ☐ Yes – where? ____
    - ☐ No

31a. **If yes:** When did you last live there? __________________
31b. **If yes:** How long did you live there? ______________

32. Since living in the United States, how many times have you traveled to places with malaria/ Africa?
    - ☐ Never – this will be my first time back
    - ☐ 1
    - ☐ 2
    - ☐ 3
    - ☐ 4
    - ☐ 5 or more times

33. Where were your parents born? (Country): Mom: _________________ Dad: ______________________

34. How would you describe your race/ethnicity? ____

35. What is the highest level of education you completed?
    - ☐ Grade school
    - ☐ Some high school
    - ☐ High school
    - ☐ Associate degree / some college / post-secondary training
    - ☐ Bachelor’s degree
    - ☐ Graduate school

36. Primary care doctors are doctors you go to for checkups and health problems that aren’t emergencies. Do you go to a primary care doctor?
    - ☐ Yes
    - ☐ No

36a. If yes, when is the last time you went to your primary care doctor?
    - ☐ Within the last month
    - ☐ Within the last 6 months
    - ☐ Within the last year
    - ☐ 1-2 years
    - ☐ More than 2 years ago

37. Additional notes: ____

POST TRAVEL FOLLOW UP SURVEY (PHONE CALL)

1. During your most recent trip did you use any of the following strategies to keep mosquitos away or stay healthy?

I'm going to list a few examples and you can respond: yes, no, or sometimes

☐ Yes ☐ No ☐ Sometimes Bed nets
☐ Yes ☐ No ☐ Sometimes Repellent / creams / sprays / lotions
☐ Yes ☐ No ☐ Sometimes Mosquito coils
☐ Yes ☐ No ☐ Sometimes Environmental cleanup i.e., removing sources of standing water
☐ Yes ☐ No ☐ Sometimes Staying indoors when mosquitoes are out
☐ Yes ☐ No ☐ Sometimes Wear long clothing
☐ Yes ☐ No ☐ Sometimes Practice good food safety / hygiene
☐ Yes ☐ No ☐ Sometimes Ensure clean water source (safe, bottled)
☐ Yes ☐ No ☐ Sometimes Educate yourself on risks of malaria before traveling
☐ Yes ☐ No ☐ Sometimes Pick where you stayed in order to avoid mosquitos (AC/screens)
☐ Yes ☐ No ☐ Sometimes Get vaccinations

Anything else? ____

2. Did you take a malaria prevention medicine during your last trip?
   - ☐ Yes → Did you finish taking the medicine after you got back? ☐ Yes ☐ No ☐ Don't recall
   - ☐ No
   - ☐ Sometimes → Did you finish taking the medicine after you got back? ☐ Yes ☐ No ☐ Don't recall

3. Did a healthcare provider write you a prescription for a malaria prevention medicine?
   - ☐ Yes
   - ☐ No

3a**.** **<u>If yes,</u>** did you pick up the medicine in the US or once you arrived to your destination?

- ☐ Picked up the medicine in the US
- ☐ Picked up the medicine in destination country

If 2 is yes/sometimes and 3 is no, clarify: Did you buy the malaria prevention medicine without a prescription once you arrived to your destination?

- ☐ Yes
- ☐ No – Explain:

  ____

4. On your last trip did any children travel with you?
   - ☐ Yes – if yes, how many? __________________ How old were they? ________________________
   - ☐ No **Skip to question 5**

4a. **<u>If yes</u>**, did the child/ren take a malaria prevention medicine during your last trip?

- ☐ Yes → Did the child finish taking the medicine after he/she got back? ☐ Yes ☐ No ☐ Don't recall
- ☐ No
- ☐ Sometimes→Did the child finish taking the medicine after he/she got back? ☐ Yes ☐ No ☐ Don't recall

4b. Did a healthcare provider write your child/children a prescription for malaria prevention medicine?
- ☐ Yes
- ☐ No

4c**. <u>If yes</u>,** did you pick up the medicine for your child/ren in the US or once you arrived to your destination?
- ☐ Picked up the medicine in the US
- ☐ Picked up the medicine in destination country

<u>If 4a is yes/sometimes and 4b is no</u>: Did you buy the malaria prevention medicine for your child/ren without a prescription once you arrived to your destination?
- ☐ Yes
- ☐ No Explain: ____

5. **Did you experience any of the following health issues during your last trip?**

☐ Yes	☐ No	☐ Don't remember	Fever	Temperature? ________________
☐ Yes	☐ No	☐ Don't remember	Headache	
☐ Yes	☐ No	☐ Don't remember	Body aches	
☐ Yes	☐ No	☐ Don't remember	Diarrhea	
☐ Yes	☐ No	☐ Don't remember	Nausea	
☐ Yes	☐ No	☐ Don't remember	Vomiting	
☐ Yes	☐ No	☐ Don't remember	Mosquito bites	
☐ Yes	☐ No	☐ Don't remember	Heat distress	
☐ Yes	☐ No	☐ Don't remember	Fainting	
☐ Yes	☐ No	☐ Don't remember	Car crash	
☐ Yes	☐ No	☐ Don't remember	Injury	Describe: _____________________
☐ Yes	☐ No	☐ Don't remember	Animal bite	Describe: _____________________

6. While you were in ______ did you go to the hospital or a clinic for any personal health issues?
   - ☐ No
   - ☐ Yes Please describe: ____

7. After you returned to the United States, did you go to the hospital or a clinic for health issues that could have been caused by your travel?
   - ☐ No
   - ☐ Yes Please describe: ____
   ____

8. Did anyone travelling with you go to the hospital or a clinic for any health issues while you were travelling?
   - ☐ No
   - ☐ Yes Please describe: ____

9. After you returned to the United States, did anyone travelling with you go to the hospital or a clinic for health issues that could have been caused by the travel?
   - ☐ No
   - ☐ Yes Please describe: ____
